# *APOE4* exacerbates glucocorticoid stress hormone-induced tau pathology via mitochondrial dysfunction

**DOI:** 10.1038/s41419-026-08543-1

**Published:** 2026-03-27

**Authors:** Qing Yu, Fang Du, Veria Puerta-Alvarado, Jeffrey H. Goodman, Clarissa L. Waites

**Affiliations:** 1 Department of Pathology and Cell Biology, Taub Institute for Research on Alzheimer’s Disease and Aging Brain, Columbia University Irving Medical Center, New York, NY USA; 2 Institute for Basic Research in Developmental Disabilities, Staten Island, NY USA; 3 Neurobiology and Behavior PhD Program, Columbia University, New York, NY USA; 4 Departments of Physiology, Pharmacology, and Neurology, SUNY Downstate Health Sciences University, Brooklyn, NY USA; 5 Department of Neuroscience, Columbia University, New York, NY USA

**Keywords:** Alzheimer's disease, Stress and resilience

## Abstract

*APOE4* is the leading genetic risk factor for Alzheimer’s disease, and chronic stress is a leading environmental risk factor. Studies suggest that *APOE4* confers vulnerability to the behavioral and neuropathological effects of chronic stress, representing a potential mechanism by which this genetic variant accelerates Alzheimer’s onset and progression. Whether and how *APOE4*-mediated stress vulnerability manifests in neurons of the hippocampus, a brain region particularly susceptible to stress and Alzheimer’s pathology, remains unexplored. Using a combination of in vivo and in vitro experiments in humanized *APOE4* and *APOE3* knockin mice and primary hippocampal neurons from these animals, we investigated whether and how *APOE4* confers sensitivity to glucocorticoids (GCs), the main stress hormones. We found that a hallmark of stress/GC-induced brain damage, tau pathology (i.e., tau accumulation, hyperphosphorylation, and spreading) is exacerbated in *APOE4* versus *APOE3* mice. Moreover, *APOE4* animals exhibit underlying mitochondrial dysfunction and enhanced glucocorticoid receptor activation in the hippocampus, factors that likely contribute to tau pathogenesis in both the presence and absence of stress/GCs. Supporting this concept, opening of the mitochondrial permeability transition pore (mPTP) drives mitochondrial dysfunction and tau pathology in *APOE4* mice, while pharmacological inhibition of the mPTP is protective against ApoE4-mediated mitochondrial damage, tau phosphorylation and spreading, and downstream hippocampal synapse loss. These findings shed light on the mechanisms of stress vulnerability in *APOE4* carriers and identify the mPTP as a potential therapeutic target for ameliorating Alzheimer’s pathogenesis in this population.

## Introduction

Alzheimer’s disease is caused by a complex interplay of genetic and environmental factors. The strongest genetic risk factor for late-onset Alzheimer’s is *APOE4*, encoding the E4 variant of the lipid transporter apolipoprotein E (ApoE) [1]. *APOE4* is carried by ~14% of the world’s population and increases Alzheimer’s disease risk by fourfold (for heterozygotes) or 12-fold (for homozygotes) compared to the risk-neutral *APOE3* variant [1]. While environmental risk factors are harder to pinpoint, multiple studies indicate that chronic stress and elevated levels of glucocorticoids (GCs), the main stress hormones, increase Alzheimer’s risk and hasten disease progression [2–4]. Indeed, prolonged psychological stress during mid-life, high work-related stress, and high ‘distress proneness’ during middle age are all reported to significantly elevate Alzheimer’s risk, while high circulating levels of GCs are associated with faster cognitive decline in Alzheimer’s subjects [2, 3, 5–7]. Stress-related neuropsychiatric disorders, particularly depression, are also linked to a higher risk of Alzheimer’s and dementia [8, 9]. Interestingly, *APOE4* carriers exhibit a higher incidence of late-life depression, anxiety, and cognitive impairment compared to carriers of the other *APOE* isoforms [10–13], and these conditions are exacerbated to a greater extent by environmental stressors [10, 14]. These findings suggest that *APOE4* confers stress vulnerability, representing a potential mechanism by which this gene accelerates Alzheimer’s disease onset and progression. Whether and how such stress vulnerability manifests at the cellular level to promote disease pathogenesis remains unclear.

Two prominent drivers of Alzheimer’s disease are mitochondrial dysfunction and tau pathogenesis (i.e., hyperphosphorylation, oligomerization, and trans-cellular spreading of tau) [4, 15, 16]. We previously showed that high GC levels strongly induce both of these features in the murine hippocampus and that they are linked, with GC-induced mitochondrial dysfunction promoting tau pathology [17]. ApoE4 expression also promotes these features. Indeed, the brains of *APOE4* carriers (humans and targeted replacement *APOE4* knockin mice) exhibit altered mitochondrial structure and function, including impaired mitochondrial fission and mitophagy, elevated levels of reactive oxygen species (ROS) and oxidative stress markers, and decreased mitochondrial membrane potential and respiration [18–21]. Interestingly, *APOE4* mice also exhibit age- and stress-induced depressive behaviors that are rescued by supplementation with ATP [20], suggesting that disruption of mitochondrial ATP production contributes to stress vulnerability in *APOE4* carriers. Moreover, *APOE4* exacerbates tau hyperphosphorylation and tau-mediated neuronal loss and brain atrophy in the PS19 tauopathy mouse model [22], and *APOE4* deletion from brain cells of these animals significantly mitigates these phenotypes [23, 24]. Collectively, these studies indicate that ApoE4 expression disrupts mitochondrial function and promotes tau pathology, although the link between these two processes in *APOE4* carriers remains unclear.

In the current study, we examined the effects of GCs on tau pathogenesis and mitochondrial function in humanized *APOE4* vs *APOE3* knockin mice. We found that GC-induced tau pathology, including tau accumulation, phosphorylation, and spreading, is exacerbated in *APOE4* vs. *APOE3* carriers. Middle-aged *APOE4* animals also exhibit oxidative stress, mitochondrial dysfunction, and enhanced GR activation in the hippocampus under control conditions, suggesting that these underlying factors contribute to tau pathogenesis in the presence and absence of GCs. Further, our findings implicate the mitochondrial permeability transition pore (mPTP) in these processes and show that mPTP inhibition is protective against both mitochondrial damage and tau pathology in *APOE4* mice. These findings shed light on the mechanisms of stress vulnerability and Alzheimer’s disease pathophysiology in carriers of the *APOE4* gene.

## Materials and methods

### Mice

Humanized APOE3 knock-in (KI) mice (strain #029018) and APOE4 KI mice (strain #027894) were obtained from The Jackson Laboratory. APOE4 KI mice were created by replacing exons 2–4 of the mouse *Apoe* gene with 1.5 kb of the human *APOE4* sequence (exons 2, 3, 4, and some 3’ UTR sequence) to create the *Apoe*^tm1.1(APOE*4)Adiuj^ allele, and APOE3 KI mice by CRISPR/Cas9-mediated introduction of the R130C point mutation into this allele [25, 26]. PS19 (strain #008169) mice [27] were also obtained from The Jackson Laboratory. All animal studies were carried out with the approval of the Columbia Institutional Animal Care and Use Committee (IACUC) in accordance with the National Institutes of Health guidelines for animal care. Animal numbers (n) for experiments were obtained based on the estimated effect sizes calculated in our previous studies [17, 28]. Offspring of transgenic (Tg) mice: APOE3( + */* + )(E3), APOE4( + */* + )(E4), PS19( + */-*)/APOE3( + */* + )(TE3), and PS19( + */-*)/ APOE4( + */* + )(TE4) mice were identified by PCR using primers for each specific transgene. Mice were administered the following drugs: DEX [D2915, Sigma; 5 mg/kg per day by intraperitoneal (IP) injection, dissolved in PBS], mito-apocynin [MitoAPO, HY-135869, MedChemExpress; 3 mg/kg per day by IP injection, dissolved in 15% polyethylene glycol (PEG400) with PBS], with 15 days DEX/mito-apocynin injection for 9–10/15–16 month-old APOE3 and APOE4 (E3 and E4) mice and 10 weeks mito-apocynin injection for 1–1.5 months PS19/APOE4 (TE4) mice. Control animals received daily intraperitoneal injections of PBS (DEX vehicle) or 15% PEG400 in PBS (mito-apocynin vehicle). Both male and female mice were used in the experiments. Mice were allocated randomly into different groups as indicated.

### Evaluation of serum corticosterone levels

Endogenous corticosterone serum levels were measured using the Corticosterone Parameter Assay kit (R&D Systems, KGE009) as described previously [17]. The wavelength for measurement was 450 nm, and the correction wavelength was 570 nm.

### Primary hippocampal culture

Primary neurons were prepared from postnatal day 0 (P0) APOE3 or APOE4 hippocampi, and maintained in 24-well plates with Neurobasal medium supplemented with B27, 600 μM L-glutamine, and antibiotic-antimycotic, as described previously [29]. At 11–12 days in vitro (DIV), hippocampal neurons were treated with vehicle, mito-apocynin (mAPO, 1 μM, HY-135869, MedChemExpress), or cyclosporin A (CsA, 1 μM, C1832, Sigma) for 1 h, and then with dexamethasone (DEX, 1 µM, D2915, Sigma) for 48 hours. For all conditions, primary neuronal cultures were collected for immunoblotting, mitochondrial function assay, or immunostaining at 13–14 DIV.

### Media preparation for immunoblot and ELISA

When indicated, the cell culture media were prepared as described previously [28, 29]. Briefly, the media was collected and concentrated using Pierce™ Protein Concentrators PES with 30 K molecular-weight cutoff (Thermo Scientific, 88531) followed by centrifugation at 2000 × *g* for 20 min. The supernatant was then subjected to sequential centrifugation steps: 30 min at 10,000 × *g*, 30 min at 21,000 × *g*, and finally 70 min at 100,000 × *g* to deplete extracellular vesicles (EVs). The remaining supernatant was used for the experiments as indicated.

### ELISA

EV-depleted media samples (50 µL volume) were used for measurement of Tau concentration by mouse-specific total Tau ELISA kit (KMB7011, Thermo Scientific) according to manufacturer’s instructions.

### Immunoblotting

Protein extracts were separated by SDS/PAGE (10% Tris-Glycine gel; XP00105BOX, Invitrogen), then transferred to nitrocellulose membranes (10600001, Amersham). After blocking in TBST buffer (20 mM Tris-HCl, 150 mM sodium chloride, 0.1% Tween-20) containing 5% (wt/vol) nonfat dry milk for 1 h at room temperature, the membrane was incubated with primary antibodies overnight at 4 °C, then with secondary antibodies for 1 h at room temperature. The following antibodies were used: AT8 (MN1020, ThermoFisher Scientific), PHF-1 (from Dr. Peter Davies), Tau5 (ab80579, Abcam), p-GR (4161S, Cell Signaling), GR (12041S, Cell Signaling), HSP70 (333800,Thermo Fisher), HSP90 (MA545102,Thermo Fisher), FKBP51 (PA1020, Thermo Fisher), anti-CypD (ab110324, Abcam), anti- KDM1/LSD1 (ab17721, Abcam), anti-Tom20 (OA241-4F3, NOVUS), anti-Tubulin (ab4074, Abcam). IRDye 800CW goat anti-mouse IgG secondary antibody (P/N: 926-32210, LI-COR), IRDye 680CW goat anti-rabbit IgG secondary antibody (P/N: 926-68071, LI-COR). Membranes were visualized by Odyssey Infrared Imager (model 9120, LI-COR Biosciences), and relative optical densities of bands were determined by Fiji/ImageJ software. Full immunoblots used in the figures of this manuscript are shown in Fig. S4.

### Immunofluorescence staining of brain slices and cultured neurons

**Fig. 1:**
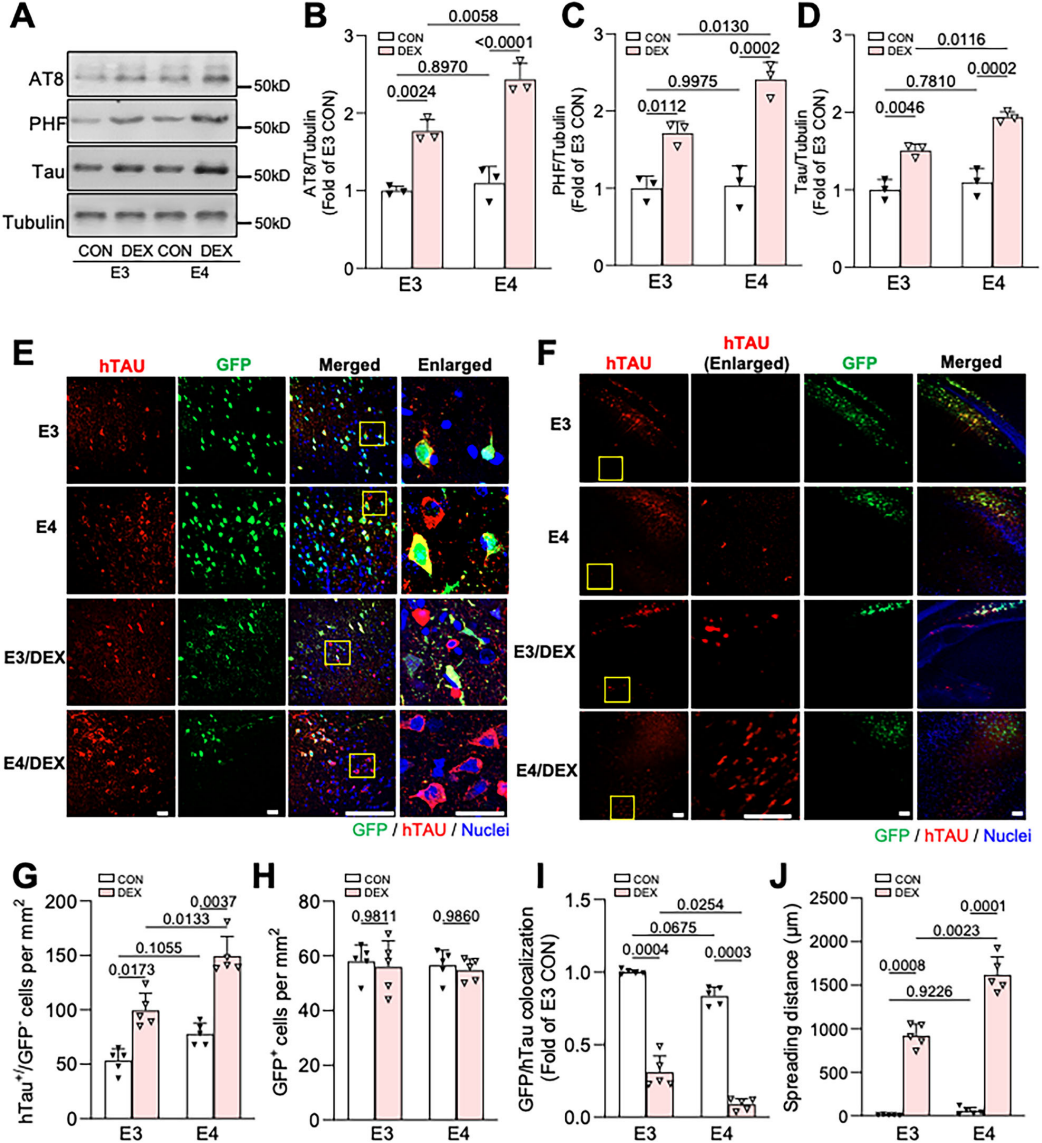
Glucocorticoid-induced tau phosphorylation and spreading are enhanced in E4 mice. **A**–**D** Representative immunoblots (**A**) and quantification (**B**–**D**) of AT8, PHF1, and total tau (Tau5) immunoreactivity in lysates from hippocampal tissue of 9–10-month-old E3/E4 mice treated with vehicle (CON) or dexamethasone (DEX). Intensity values are expressed relative to tubulin and normalized to the E3 CON condition (*P* values indicated on graphs; data presented as mean ± SD; two-way ANOVA with Tukey’s multiple comparisons test; *n* = 3 mice/condition for (**B**–**D**)). **E** Representative images showing hTau (red) and GFP (green) in CA1 neurons of mice treated as indicated. Nuclei are stained with DAPI (blue). The right column shows enlarged regions (indicated by yellow boxes). Scale bars, 50 µm. **F** Representative images depicting the spreading of hTau (red) from GFP^+^ cells near the injection site in mice treated as indicated. Yellow boxes indicate enlarged regions. Scale bars, 200 µm. **G** Quantification of hTau^+^/GFP^−^ cells per mm^2^ in mice treated as indicated. **H** Quantification of GFP^+^ cells per mm^2^ in mice treated as indicated. **I** Quantification of the GFP/hTau colocalization ratio in each condition, normalized to E3 CON condition. **J** Quantification of Tau spreading distance (mm) for each condition. **G**–**J** *P* values are indicated on graphs; data presented as mean ± SD; two-way ANOVA with Tukey’s multiple comparisons test; *n* = 5 mice/group. Each point represents an individual mouse.

Mice were perfused using 4% paraformaldehyde, and brains were then immersion-fixed for 48 h at 4 °C. Floating brain sections or fixed primary neurons were immunostained as previously described [17]. Briefly, fixed neurons or slices cut at 35 μm on a vibratome (VT1000S; Leica) Floating brain sections or fixed primary neurons were incubated overnight with the following primary antibodies: mouse anti-oligomeric Tau antibody TOMA-1 (1:2500, Millipore sigma, MABN819), mouse anti-Synapsin I antibody (1:1000, 611393, BD Biosciences), mouse anti-phospho-Tau pSer202/Thr205 (1:1000, MN1020, ThermoFisher Scientific), and chicken MAP2 (1:5000, ab5392, Abcam). They were then incubated for 1 h with secondary antibodies (Alexa Fluor 488, 594, and 633 goat anti-rabbit or anti-mouse IgG, 1:2000 dilution). Coverslips were mounted with VectaShield (Vector Laboratories) and sealed with clear nail polish. Images were acquired with a ×40 objective (Neofluar, NA 1.4) on a Zeiss LSM 800 confocal microscope running Zen2 software or with a 2× objective on an Eclipse 90i dual laser-scanning confocal microscope (NIKON, for lower magnification images in Figs. 1F, 8B). The images were manually measured and quantified using the auto-threshold settings in Fiji/ImageJ software.

### Mitochondrial/cytosolic/nuclear isolation

Mitochondrial isolation was performed using the Mitochondrial extraction kit (Novus, NBP2-29448). Briefly, tissues were washed using ice-cold 1× PBS, then 5 ml of ice-cold homogenization buffer (provided by the kit) was added per gram of tissue. Following homogenization in a Dounce-type homogenizer, cell suspensions were transferred to tubes and nuclear pellets collected following centrifugation at 700× *g* for 10 min at 4 °C. The nuclei pellets were resuspended in fractionation buffer (20 mM HEPES, pH 7.4, 10 mM KCl, 2 mM MgCl_2_, 1 mM EDTA, 1 mM EGTA, 1 mM DTT, and PI cocktail) and vigorously rocked for 30 min at 4 °C. Nuclear extracts were collected after centrifugation for 10 min at 700× *g* (4 °C). The cytosolic fraction (supernatant) and the mitochondrial pellet were collected separately according to the manufacturer’s instructions.

### Mitochondria functional assays

Complex I activity and ATP production were measured from 13 to 14 DIV hippocampal neurons or hippocampal tissue with Complex I Enzyme Activity Microplate Assay Kit (ab109721, Abcam) and ATP Assay Kit (Colorimetric/Fluorometric, ab83355, Abcam), respectively, according to the manufacturer’s instructions.

### Evaluation of mitochondrial and cerebral reactive oxygen species

To visualize the production of reactive oxygen species (ROS), fresh, unfixed brain sections from mouse hippocampal tissue or primary hippocampal neurons were incubated with 1 μM MitoSOX Red (M36008, ThermoFisher), a fluorochrome specific for anion superoxide produced in the inner mitochondrial compartment, at 37 °C for 30 min. After MitoSOX incubation, tissue/neurons were then fixed with 4% paraformaldehyde as described above, and immunostained with anti-oligomeric Tau antibody TOMA-1 as described previously [17]. Images were acquired at 37 °C with a ×40 objective (Neofluar, NA 1.4) on a Zeiss LSM 800 confocal microscope running Zen2 software. Quantification of staining intensity and the percentage of area occupied by MitoSOX was measured and quantified using the auto-threshold settings in Fiji/ImageJ software.

Intracellular ROS levels were measured by electron paramagnetic resonance (EPR) spectroscopy. Here, fresh brain tissues were incubated with CMH (cyclic hydroxylamine 1-hydroxy-3-methoxycarbonyl-2, 2, 5, 5-tetramethyl-pyrrolidine, 100 μM) for 30 min, then washed three times with ice-cold PBS. The tissues were collected and homogenized with 100 μl of PBS for EPR measurement. EPR spectra were collected, stored, and analyzed via electron paramagnetic resonance (EPR, Bruker ESR 5000, Germany) [30].

### Evaluation of mPTP opening

Hippocampal neurons (1 × 10^2^ cells/well, DIV 13–14) plated onto Lab-Tek 4-well chamber slides were treated with 1 μM Calcein Green AM (C3099, Thermo Fisher Scientific) at 37°C for 30 min, then treated ±1 mM cobalt chloride (CoCl_2_) for 30 min. Images were acquired at 37 °C with a 40X oil-immersion objective (Neofluar, NA 1.3) on an epifluorescence microscope (Axio Observer Z1, Zeiss) with Colibri LED light source, EMCCD camera (Hamamatsu), and Zen 2012 (blue edition) software. Quantification of calcein fluorescence intensity was measured and quantified using the auto-threshold settings in Fiji/ImageJ software.

### AAV injection procedure

Details of this procedure are described in previous studies [28, 29]. Briefly, AAV.CBA.eGFP.2 A.P301L-Tau plasmid (Addgene plasmid #140425) was packaged into AAV8 serotype. Prior to AAV injection, male/female mice (5/group) were administered DEX (5 mg/kg, i.p. injection) or mito-apocynin (3 mg/kg, i.p. injection) for 7 days. Stereotactic AAV injections were performed under standard aseptic surgery conditions as previously described [28, 31]. Mice were anaesthetized with isoflurane (2%), placed in a stereotactic frame (digital stereotaxic device, Stoelting Co.), and injected bilaterally with 2 μl of AAV in hippocampal region CA1 (at the following coordinates relative to Bregma: A/P − 2.7 mm, M/L ± 2 mm, D/V − 1.5 mm) with a 10 μl Hamilton syringe at a rate of 0.25 μl/min by a Nano-injector system (Stoelting microsyringe pump, Stoelting Co.). Afterwards, the skin over the injection site was sutured, and mice were placed on a warming pad during their recovery. Mice were then administered DEX or mito-apocynin for an additional 14 days prior to euthanasia. Control animals received daily i.p. injections of PBS (DEX vehicle) or 15% PEG400 in PBS (mito-apocynin vehicle).

### Quantification of Tau spreading

hTau^+^ neurons (detected by immunostaining with Tau13 antibody) were counted in hippocampi of coronal brain sections (sectioned at 100 mm using a vibratome) near the site of AAV injection, identified by the dense cluster of GFP^+^ neurons. Tau spreading was quantified as in previous studies, including ours [28, 31, 32], by calculating the number of hTau^+^ neurons in the hippocampus that did not exhibit GFP fluorescence (hTau^+^/GFP^−^ neurons) per mm^2^ and the fraction of hTau^+^ cells that were GFP^+^ (GFP/hTau colocalization). For each condition, we also measured the maximum distance between hTau^+^ neurons in the vicinity of the hippocampal formation and the cluster of GFP^+^ neurons near the injection site, using the Fiji/ImageJ measurement tools.

### Statistical analysis

All values were expressed as the mean ± SEM. All graphing and statistical analyses were performed using GraphPad Prism (GraphPad Prism10.Ink). Statistical details of experiments are provided in the figure legends. Statistical analyses were performed with unpaired, two-tailed *t* test, or with one-way or two-way ANOVA with Tukey’s test for multiple comparisons. Values of *P* < 0.05 were considered statistically significant. Investigators were blinded to treatment conditions when performing analyses for all experiments.

### Clustering, differential expression gene analysis, and functional ontology

The RNASeq dataset analyzed is from Zalocusky et al. [33] and can be found in the Gene Expression Omnibus (GEO) under GSE167497. Raw gene matrices in the supplementary file under each 10-month-old sample (four samples each for APOE3-KI and APOE4-KI) were downloaded in May 2025, totaling eight samples. Data was formatted and clustered using Seurat [34] v5.3.0. Each sample was made into its own Seurat object, then merged with project notes detailing which KI group (E3/E4) and sample it was. Data was filtered to exclude low-quality samples and dublets/multiplets. Data were filtered to only include cells with 3500–200 genes, 10,000–500 UMIs, and <0.05% mitochondrial reads. Antisense genes and pseudogenes were also filtered out. This filtering resulted in a final matrix of 27,500 genes by 45,682 nuclei. Data was normalized with the Seurat function NormalizeData with a factor of 10,000. After performing PCA, clustering was done using Seurat functions: FindNeighbors and FindClusters, with 19 dimensions and 0.6 resolution. Dentate Gyrus granule cells clusters 1 and 2 were identified through DotPlot and FeaturePlot using these markers: *Prox1, Pdzd2, Tox3, Stxbp6, Dgkh*. Data was then formatted for removing unwanted variation (RUVs) [35] v1.40.0 and EdgeR [36] v4.4.2 differential gene expression analysis. For RUVs, a *k* of 2 was used to separate the samples by experimental condition (E3 or E4 KI) for both clusters. A false discovery rate of 0.20 was then used for generating the differentially expressed gene lists using EdgeR. We chose to use a higher FDR due to the small sample size and other clusters’ DEG analysis showing previously reported AD-relevant genes around the 0.20 FDR cutoff. After generating the upregulated and downregulated DEG lists for both cluster 1 & 2, we input them into ShinyGO [37] v0.82 using an FDR cutoff of 0.10 and species Mus musculus (GRCm39, 10090 ENSEMBLE). We also uploaded to ShinyGO the background gene list of the initial 27,500 genes detected from the raw data before clustering. All relevant gene lists (upregulated, downregulated, background, etc.) are in Supplemental Table 1.

### Ethics approval and consent to participate

All animal studies were carried out with the approval of the Columbia University Irving Medical Center Institutional Animal Care and Use Committee (CUIMC IACUC), under protocol # AC-AABK1555, and in accordance with the National Institutes of Health Guidelines for Laboratory Animal Care, recommendations from the *Guide for the Care and Use of Laboratory Animals* prepared by the National Research Council, and the American Veterinary Medical Association Guidelines on Euthanasia.

## Results

### Glucocorticoid-induced tau pathology is amplified by *APOE4*

To determine whether the *APOE4* genetic variant exacerbates GC-induced brain pathology, we first assessed tau accumulation and phosphorylation in middle-aged (9-10-month-old) humanized *APOE3* (E3) and *APOE4* (E4) knockin mice obtained from The Jackson Lab [25, 26]. Animals were administered vehicle or the synthetic GC dexamethasone (DEX; 5 mg/kg daily, i.p. injection) for 15 days, a widely-used treatment that mimics the high circulating GC levels induced by chronic stress [17, 38–41]. DEX administration had similar effects on body weight loss and suppression of endogenous corticosterone levels in both genotypes (Fig. S1A, B), indicative of their equivalent physiological responses to GCs. Following brain tissue harvest, we measured the levels of total tau and phospho-tau epitopes commonly associated with tauopathies such as Alzheimer’s by immunoblotting hippocampal lysates with Tau5 (total tau), PHF-1 (S396/S404), and AT8 (S202/T205) antibodies. We saw no difference in total or phosphorylated tau levels in lysates from vehicle-treated E4 versus E3 mice (Fig. 1A–D), consistent with a previous report that E4 targeted replacement animals lack overt tau pathology at 9 months of age [42]. DEX treatment significantly increased total and phospho-tau levels in both E3 and E4 genotypes, but caused a more dramatic increase in tau accumulation in the E4 hippocampi (~2.5-fold vs. ~1.5-fold; Fig. 1A–D). Since tau phosphorylation is known to stimulate its secretion [43, 44], and we recently found that GCs promote tau secretion and spreading [28], we also evaluated GC-induced tau spreading in E3 and E4 mice. Here, 9–10-month-old animals were pre-treated with vehicle or DEX for 7 days, then injected in the hippocampal area CA1 with adeno-associated virus (AAV) co-expressing GFP and the human frontotemporal dementia-associated tau mutant P301L (hTau) separated by the 2A cleavage sequence (AAV.CBA.eGFP.2 A.P301L-Tau) [32], enabling visualization of trans-cellular tau spreading. Animals were then administered vehicle or DEX for an additional 14 days, followed by brain tissue harvest and immunostaining with antibodies against hTau (Fig. 1E, F). Tau propagation was quantified as previously described [31, 32], by calculating hTau^+^/GFP^−^ neurons per mm^2^, GFP/hTau colocalization, and maximal spreading distance of hTau from GFP^+^ neurons (Fig. 1G–J). These studies revealed that while AAV transduction efficiency (number of GFP^+^ cells per mm^2^) was equivalent between genotypes (Fig. 1H), tau propagation was significantly enhanced in DEX-treated E4 versus E3 animals, based on the increased number of hTau^+^/GFP^-^ neurons, decreased GFP/hTau colocalization, and increased hTau spreading distance in E4 hippocampus (~1500 μm vs. ≤1000 μm in the E3 background; Fig. 1G, I, J). Together, these findings demonstrate that GC-induced tau accumulation and spreading are significantly exacerbated by the *APOE4* variant.

### GCs augment baseline mitochondrial dysfunction in E4 mice

**Fig. 2:**
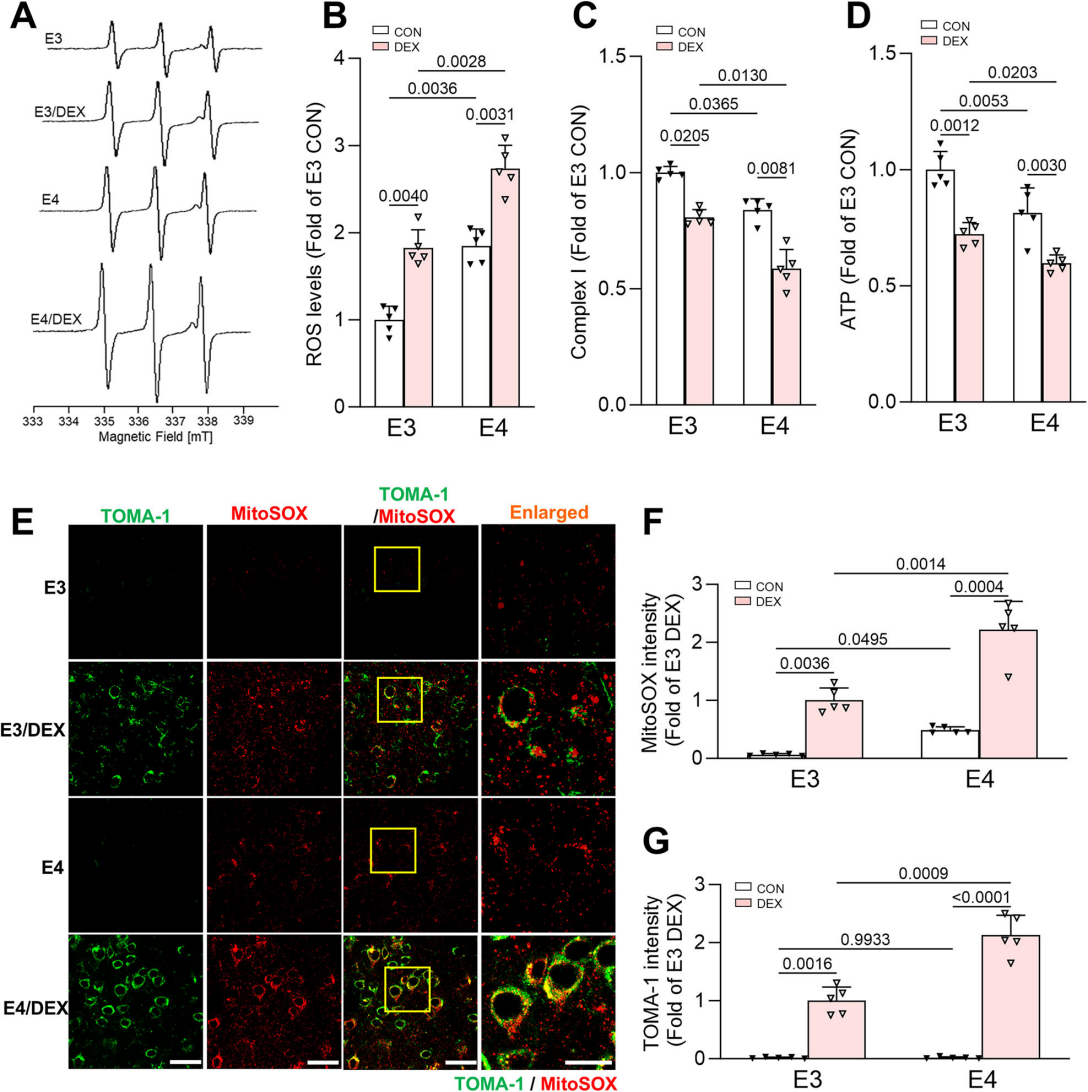
Underlying mitochondrial dysfunction amplifies the effect of GCs in E4 mice. **A**, **B** Representative spectra from electron paramagnetic resonance (EPR) spectroscopy of hippocampal tissue from 9 to 10-month-old E3 and E4 mice treated with vehicle (CON) or dexamethasone (DEX)(**A**), and accompanying quantification (**B**). Peaks in the spectra represent ROS levels (*P* values indicated on graphs; data presented as mean ± SD; two-way ANOVA with Tukey’s multiple comparisons test; *n* = 5 mice/condition). **C**, **D** Complex I activity (**C**) and ATP levels (**D**) in hippocampal tissue from E3 and E4 mice treated as indicated, normalized to the E3 CON condition (*P* values indicated on graphs; data presented as mean ± SD; two-way ANOVA with Tukey’s multiple comparisons test; *n* = 5 mice/condition). **E**–**G** Representative images (**E**) and quantification (**F**, **G**) of MitoSOX and TOMA-1 fluorescence intensity in slices from hippocampal area CA1 of mice treated as indicated. Right-most column shows enlarged regions (indicated by yellow boxes). Scale bars = 50 µm. Intensity values are normalized to the E3 DEX condition (*P* values indicated on graphs; data presented as mean ± SD; two-way ANOVA with Tukey’s multiple comparisons test; *n* = 5 mice/condition). Each point represents an individual mouse.

We recently showed that GC-induced tau pathology is precipitated by mitochondrial damage [17]. ApoE4 expression is associated with mitochondrial dysfunction in humans, mice, and cell culture models [18–21, 42, 45–47], and may increase mitochondrial susceptibility to GC-mediated damage, thus promoting more extensive downstream tau pathology. To investigate this possibility, we first measured reactive oxygen species (ROS) levels, an indicator of mitochondrial dysfunction, using highly sensitive electron paramagnetic resonance (EPR) spectroscopy on hippocampal tissue of the 9–10-month-old vehicle- and DEX-treated E3 and E4 animals described above (Fig. 1). We saw a nearly twofold increase in ROS levels in control E4 versus E3 tissue (Fig. 2A, B), indicative of underlying oxidative stress in E4 hippocampi. Moreover, DEX treatment increased ROS to a greater extent in the E4 samples, suggesting mitochondrial vulnerability to GCs. To follow up on these findings, we evaluated mitochondrial function in this tissue more directly by measuring the activity of complex I, the first component of the mitochondrial electron transport chain, and ATP production. Interestingly, both complex I activity and ATP production were significantly reduced in E4 compared to E3 tissue under control conditions, and both were inhibited to a greater extent by DEX treatment in the E4 background (Fig. 2C, D). We further monitored the impact of DEX on mitochondrial ROS production in E3 and E4 hippocampi using the fluorescent superoxide sensor MitoSOX. Here, we found that mROS levels were higher at baseline in E4 vs. E3 hippocampal tissue, and also more dramatically increased by DEX (Fig. 2E, F). Since we previously showed that oligomeric tau is closely associated with mROS in hippocampal neurons [17], we also measured oligomeric tau levels in these hippocampal slices with TOMA-1 antibodies (Fig. 2E, G). Similar to its impact on mROS, we found that DEX treatment increased oligomeric tau to a greater degree in the E4 versus the E3 background, and that this oligomeric tau colocalized with mROS in neuronal cell bodies (Fig. 2E, G). These data suggest that underlying mitochondrial dysfunction aggravates GC-driven mitochondrial damage and subsequent tau oligomerization in E4 animals.

### GR activation is enhanced in *APOE4* carriers

**Fig. 3:**
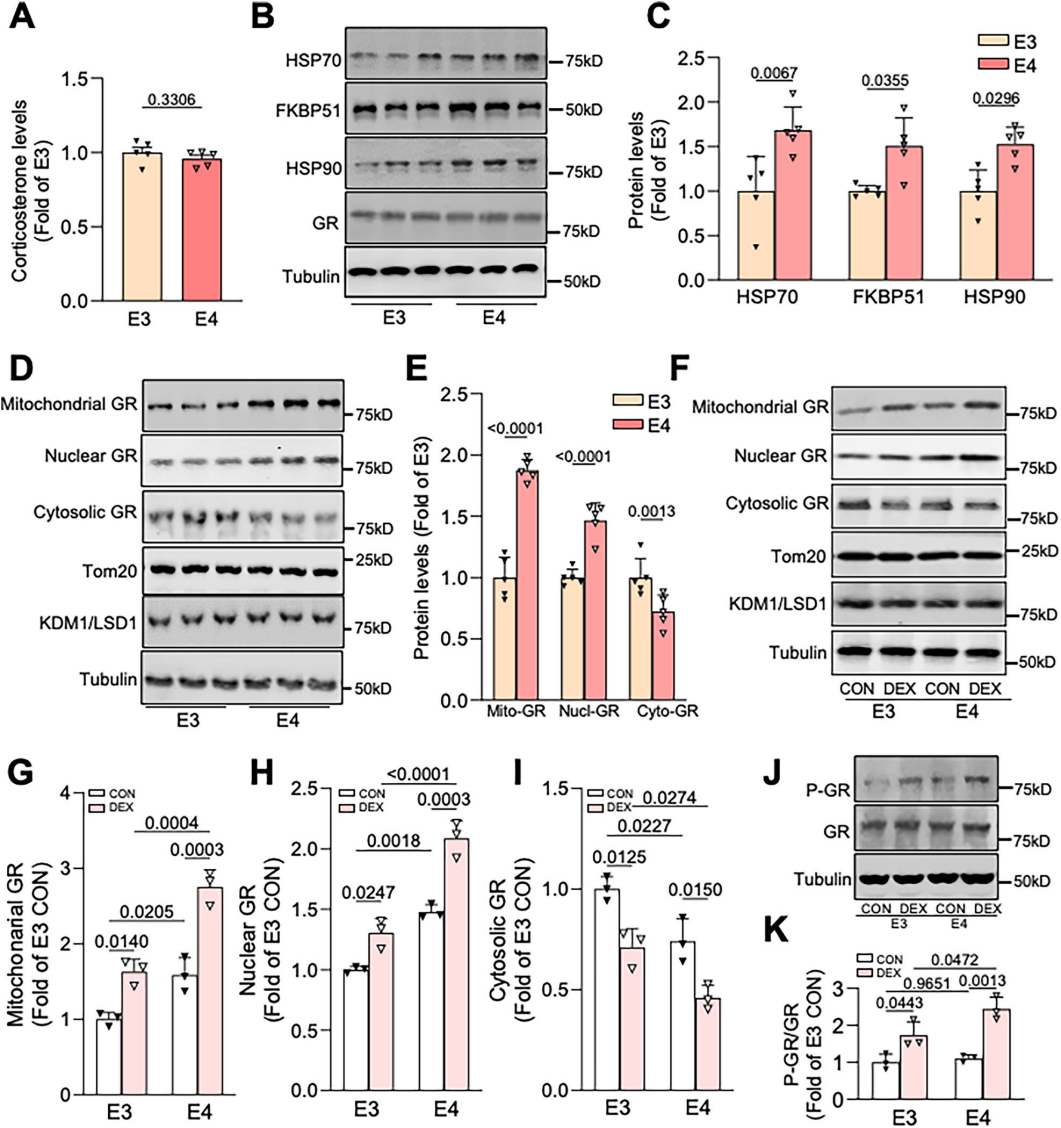
GR activation is enhanced in hippocampal tissue from E4 animals. **A** Quantification of corticosterone levels in plasma from 9 to 10-month-old E3/E4 mice, measured by ELISA (*P* values indicated on graphs; data presented as mean ± SD; unpaired *t* test; *n* = 5 mice/condition). **B**, **C** Representative immunoblots (**B**) and quantification (**C**) of Hsp70, FKBP51, and Hsp90 immunoreactivity in hippocampal lysates from the indicated mice. Intensity values are expressed relative to tubulin and normalized to the E3 group (*P* values indicated on graphs; data presented as mean ± SD; unpaired *t* test; *n* = 5 mice/condition). **D**, **E** Representative immunoblots (**D**) and quantification (**E**) of mitochondrial, nuclear, and cytosolic GR, based on immunoreactivity from mitochondrial, cytosolic, and nuclear fractions of the indicated mice. Intensity values are expressed relative to mitochondrial marker TOM20, nuclear marker KDM1/LSD1, and cytosolic marker tubulin, and normalized to the E3 group (*P* values indicated on graphs; data presented as mean ± SD; unpaired *t* test; *n* = 5 mice/condition). **F**–**I** Representative immunoblots (**F**) and quantification (**G**–**I**) of mitochondrial, nuclear, and cytosolic GR, based on immunoreactivity from mitochondrial, cytosolic, and nuclear fractions of 9–10-month-old E3/E4 mice treated with vehicle (CON) or DEX. Intensity values are expressed relative to mitochondrial marker TOM20, nuclear marker KDM1/LSD1, and cytosolic marker tubulin, and normalized to the E3 CON group (*P* values indicated on graphs; data presented as mean ± SD; two-way ANOVA with Tukey’s multiple comparisons test; *n* = 3 mice/condition). **J**, **K** Representative immunoblots (**J**) and quantification (**K**) of phospho-GR levels, normalized to total GR, in hippocampal lysates from 9 to 10-month-old E3/E4 mice +/− dexamethasone (DEX). Intensity values are expressed relative to tubulin and normalized to the E3 CON group (*P* values indicated on graphs; data presented as mean ± SD; two-way ANOVA with Tukey’s multiple comparisons test*;* *n* = 3 mice/condition). Each point represents an individual mouse.

GCs signal through their binding to glucocorticoid receptors (GRs), ligand-dependent transcription factors that regulate gene expression via binding to glucocorticoid response elements of target genes [48]. GRs are primarily localized to the cytoplasm of cells, but in response to GC binding they translocate to the nucleus and/or mitochondria to mediate transcription of nuclear and/or mitochondrial DNA [48]. GR ligand binding and activation are regulated by interactions with its molecular chaperones Hsp90, Hsp70, and FKBP51/52 [48, 49]. In particular, Hsp90 has a role in GR activation [49, 50], and Hsp90 mRNA was found to be upregulated in neurons and glia carrying *APOE4* [23], suggesting that ApoE4 expression may enhance GR activation and signaling. To explore this concept, we measured the levels of plasma corticosterone, the endogenous rodent GC, by ELISA, and of Hsp90, Hsp70, and FKBP51 by immunoblotting hippocampal lysates from 9 to 10-month-old E3 and E4 mice. Indeed, while global corticosterone levels were indistinguishable between genotypes, levels of all three co-chaperones were significantly elevated in E4 versus E3 tissue (Fig. 3A–C), suggesting that GR activation could be altered in E4 brains. As a proxy for GR activation/signaling, we next examined GR levels in cytoplasmic, nuclear, and mitochondrial fractions isolated with high purity from hippocampal tissue of these animals, as evidenced by immunoblotting with the mitochondria-specific marker Tom20, the cytoplasm-enriched marker tubulin, and the nuclear-enriched marker KDM1/LSD1 (Fig. S1C). Consistent with the other findings, we observed significantly increased nuclear and mitochondrial GR levels, and concomitantly decreased cytoplasmic GR levels, in lysates from E4 compared to E3 animals (Fig. 3D, E), indicative of increased GR activation and translocation into the nucleus and mitochondria. Further, we found that nuclear and mitochondrial GR levels were significantly increased, and cytoplasmic GR levels significantly decreased, in DEX-treated E4 versus E3 animals, and that the magnitude of DEX-induced GR translocation into the nucleus and mitochondria was also increased in E4 versus E3 animals (Fig. 3F–I). In addition, we measured GR phosphorylation, another indicator of GR activation, in hippocampal lysates from the DEX-treated E3 and E4 mice. We found that phospho-GR levels were more significantly elevated by DEX in E4 hippocampus (Fig. 3J, K), consistent with the concept that ApoE4 induces cellular changes that lower the threshold of activation for GR. Finally, using a published RNASeq dataset [33], we assessed which Gene Ontology Biological Processes (GO-BP) were present and relevant to GC synthesis and secretion in hippocampal dentate granule cells of 10-month-old APOE3 and APOE4 KI mice (Fig. S2A–E and Table 1). Functional enrichment analysis revealed significant (FDR < 0.05) downregulation of genes in the GO-BP terms “negative regulation of corticosterone secretion” and “negative regulation of GC secretion” (Fig. S2G and Table 1), indicating a decrease of factors that inhibit GC signaling. Our analysis also revealed GO-BP terms indicating potential upregulation of GCs (e.g., response to GC, growth hormone receptor signaling pathway; FDR < 0.18; Fig. S2F and Table 1). Altogether, these findings provide evidence that *APOE4* carriers exhibit enhanced GR/GC signaling at baseline and a lower threshold for GR activation by GCs.

**Table 1:** Glucocorticoid-relevant DEGs in the dentate gyrus of *APOE4* mice.

Upregulated dentate gyrus glucocorticoid-relevant pathways*						
Enrichment FDR	nGenes	Pathway genes	Fold enrichment	Pathway	URL	Genes
0.039199256	15	1534	2.893214048	GO:0007267 cell–cell signaling	http://amigo.geneontology.org/amigo/term/GO:0007267	Grm3 Glra2 Mctp1 Grm8 Tnks Gpc3 Prkcg Scn1b Slc6a1 Shc3 Adcy8 Zfp423 Ptprn2 Dcc Grp
0.083726719	6	320	5.532120109	GO:0007611 learning or memory	http://amigo.geneontology.org/amigo/term/GO:0007611	Slc6a1 Kcnk10 Camk4 Prkcg Adcy8 Shc3
0.116656952	2	17	34.0605042	GO:0060396 growth hormone receptor signaling pathway	http://amigo.geneontology.org/amigo/term/GO:0060396	Leprotl1 Ghr
0.171040136	4	179	6.57987013	GO:0051384 response to glucocorticoid	http://amigo.geneontology.org/amigo/term/GO:0051384	Bcl2 Pam Slit2 Ghr
Upregulated Dentate Gyrus DEG list**						
Cluster 1 Upregulated DEG List			Gm15594 Slit2 Serpina3n D8Ertd82e Gm20404 Zfp804b Leprotl1 Ralgapa2 Tnks Erdr1 Timp4 Aim2 Pam Cpne4 Kcnb2 Samd4 Lrrtm4 Gpc3 Rad51ap2 Mlip Lsm14a Grm8 Grp Scn1b Bcl2 Ptprn2 Plekhh2 Prkcg 4930555F03Rik Unc5d Hmgcll1 Tenm2 Ghr Camk4 Arhgap6 Ptchd4 Dach1 Glra2 Rccd1 Robo2 Tmc4 Nufip2 Shc3 Dcc Esyt2 Laptm4a Pitpnm3 Rpa3 Tenm3			
Cluster 2 Upregulated DEG List			Gm15594 Gm20404 Zfp804b Leprotl1 Smim18 D8Ertd82e Gpc3 Rad51ap2 3632451O06Rik Pcsk6 Rpa3 Tmc4 Leng1 Aim2 Slit2 Npas3 Cntn5 Zfp423 Cpne4 Eln Sgcz Timp4 Grm3 Adcy8 Samd4 Lrrtm4 BC030499 2210408I21Rik Kcnb2 Gm29242 Plekhh2 Ralgapa2 Gm14582 Cnbd1 A630012P03Rik Erdr1 Arhgap6 Gpr158 Lsm14a Rapgef5 Mctp1 Robo2 Trim71 Fam19a1 Sntg2 Pabpn1 Olfm3 Pcdh11x Scn1b Mlip Pam Itm2c Wdhd1 Glra2 Slc6a1 Uckl1os Tenm2 Plce1 Kcnk10 Mkx			

Downregulated dentate gyrus pathways						
Enrichment FDR	nGenes	Pathway genes	Fold enrichment	Pathway	URL	Genes
0.022705932	2	3	157.1007752	GO:2000853 negative regulation of corticosterone secretion	http://amigo.geneontology.org/amigo/term/GO:2000853	Nrg1
0.031275448	2	6	78.5503876	GO:2000832 negative regulation of steroid hormone secretion	http://amigo.geneontology.org/amigo/term/GO:2000832	Nrg1
0.031275448	2	6	78.5503876	GO:2000847 negative regulation of corticosteroid hormone secretion	http://amigo.geneontology.org/amigo/term/GO:2000847	Nrg1
0.031275448	2	6	78.5503876	GO:2000850 negative regulation of glucocorticoid secretion	http://amigo.geneontology.org/amigo/term/GO:2000850	Nrg1
0.058598798	2	12	39.2751938	GO:0035331 negative regulation of hippo signaling	http://amigo.geneontology.org/amigo/term/GO:0035331	Limd1 Cit
0.058598798	2	14	36.25402504	GO:2000849 regulation of glucocorticoid secretion	http://amigo.geneontology.org/amigo/term/GO:2000849	Nrg1
0.063031548	2	16	33.66445183	GO:0035933 glucocorticoid secretion	http://amigo.geneontology.org/amigo/term/GO:0035933	Nrg1
0.065496371	2	16	31.42015504	GO:0033194 response to hydroperoxide	http://amigo.geneontology.org/amigo/term/GO:0033194	Rnf112 Prkd1
0.086028142	3	69	10.71141649	GO:0060986 endocrine hormone secretion	http://amigo.geneontology.org/amigo/term/GO:0060986	Pparg Nrg1
0.10559898	4	162	5.928331139	GO:0007613 memory	http://amigo.geneontology.org/amigo/term/GO:0007613	Plk2 Grm7 Nrg1

**Downregulated Dentate Gyrus DEG list**						
**Enrichment FDR**	**nGenes**	**Pathway genes**	**Fold enrichment**	**Pathway**	**URL**	**Genes**
Cluster 1 Downregulated DEG List			Nrg1 Gm44151 Gm10600 Tex21 Tia1 Mov10l1 Gabra2 Gm16867 Gm44257 Rgs6 Fbxl7 Chst9 Opcml Gm12576 4921539H07Rik Spon1 Gm26905 St7 Gm12394 Chrm3 Lmod1 Cttnbp2 Sycp3 Add2 Gm2163 Ermard Serinc2 Gm17231 Slc24a3 Mbd4 Akap12 Gm5087 Grm7 Rph3al Rimbp2 Dync2h1 Micu3 Glis3 Kif17 Gm3448 Elmod2 A330102I10Rik Ppp6r1 Rnf112 Fam135b A330015K06Rik Arl15 Gm42477 Myt1l Grip1 Mios Slc7a15 Hspa5 Dtx3 Kxd1 Mycbp2 B4galt1 Mtmr4 Fdft1 Zfp462 Sycp2 4933411O13Rik Glp2r Edil3 Mmrn2 Pdia4 P2ry14 Kcnc2 Prkd1 St6galnac3			
Cluster 2 Downregulated DEG List			Gm10600 Nrg1 Mov10l1 Gm44151 Loxl2 Tia1 Tex21 Gm12394 Rgs6 Gm44257 Gm12576 Cttnbp2 St7 Gm2163 Serinc2 Gabra2 Akap12 St6galnac3 Slc15a2 Add2 Sycp3 Dpf3 Gm43154 Ermard Lhfp H2afz Hspa5 Gm5087 Slc38a11 Cntfr Gm17231 Myt1l Pparg Plk2 Nrxn3 Mfsd12 Fbxl7 Grm7 A330102I10Rik Sycp2 4930578G10Rik 4921539H07Rik Kbtbd11 Oprd1 Tmem191c Gm11713 Glp2r B4galt1 Pacsin2 Kcna1 Taf7l Lyn Reps2 Mbd4 Cit Opcml Ptprg Gm3448 Fstl4 Rimbp2 Gm42477 Parp8 Ank1 Ormdl2 Limd1 Rph3al Tra2a Shroom4 Sel1l Rgs11			

*These pathways were selected because of their relevance to GR activation/glucocorticoid pathways, determined by inputting a GC-relevant gene list against the same background gene list used for these pathways (see “Methods”). For a full list of pathways, see the attached supplementary Table file. All pathways were generated using ShinyGO v0.82, GO Biological Process, FDR = 0.10.

Table Headers: “Enrichment FDR” stands for the False Discovery Rate for the Fold Enrichment values and statistical significance can be determined, similar to *P* values (FDR < 0.05), although FDRs <0.01 or 0.001 are preferred with larger gene sets. “nGenes” refers to the number of genes from the combined DEG list of both cluster 1 and 2 (duplicate genes were removed) that are associated with the pathway. “Pathway Genes” refers to the total number of genes belonging to that pathway, which is independent of our DEG lists. “Fold Enrichment” refers to how overrepresented the pathway genes are in proportion to the rest of the genes from the background.

**This is the complete differentially expressed genes (DEGs) list from both clusters when using an FDR of 0.20. Genes are in order from highest statistical significance to least. For the FDRs, *P* values, logFC, and logCPM of each gene, see the attached supplementary Table file.

List of differentially expressed genes relevant to GR activation/glucocorticoid pathways that are upregulated or downregulated in the dentate gyrus of APOE4 vs. APOE3 mice. Details about genes, pathways, and how they were selected are included in the Table.

**Fig. 4:**
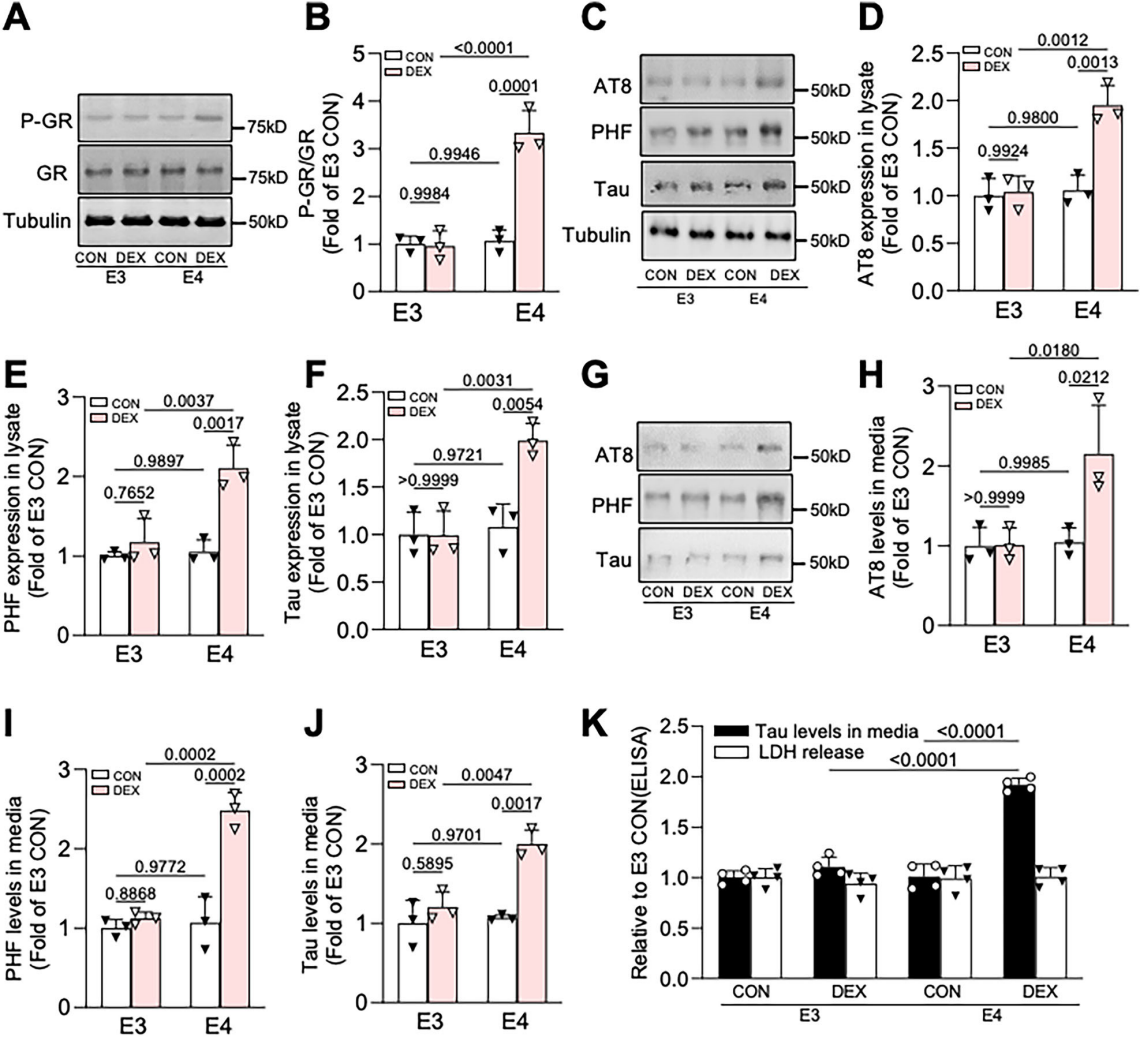
Low GC levels induce tau pathogenesis in E4 but not E3 hippocampal neurons. **A**, **B** Representative immunoblots (**A**) and quantification (**B**) of phospho-GR levels, normalized to total GR, in lysates from 12 DIV E3/E4 hippocampal neurons treated with vehicle (CON) or dexamethasone (DEX) for 48h. Intensity values are expressed relative to GR and normalized to the E3 CON group (*P* values indicated on graphs; data presented as mean ± SD; two-way ANOVA with Tukey’s multiple comparisons test; *n* = 3 samples/condition). **C**–**F** Representative immunoblots (**C**) and quantification (**D**–**F**) of AT8, PHF1, and total tau (Tau5) immunoreactivity in lysates from the indicated treatment conditions. Intensity values are expressed relative to tubulin and normalized to the E3 CON condition (*P* values indicated on graphs; data presented as mean ± SD; two-way ANOVA with Tukey’s multiple comparisons test; *n* = 3 samples/condition). **G**–**J** Representative immunoblots (**G**) and quantification (**H**–**J**) of AT8, PHF1, and total tau (Tau5) immunoreactivity in extracellular vesicle (EV)-depleted media from the indicated treatment conditions. Intensity values are normalized to the E3 CON condition (*P* values indicated on graphs; data presented as mean ± SD; two-way ANOVA with Tukey’s multiple comparisons test; *n* = 3 samples/condition). **K** Quantification of ELISA for total tau levels (black bars) and LDH release (white bars) in extracellular vesicle (EV)-depleted media from the indicated treatment conditions (*P* values indicated on graphs; data presented as mean ± SD; one-way ANOVA with Tukey’s multiple comparisons test; *n* = 4 samples/condition).

To further investigate whether ApoE4 augments neuronal responsiveness to GC/GR signaling, we treated 12-day in vitro (DIV) primary hippocampal neurons from E3 and E4 mice for 48 h with 200 nM DEX, a concentration five times lower than that which we typically use for our in vitro experiments (1 μM). Treatment with 200 nM DEX did not alter phospho-GR levels in E3 neurons, but increased these levels by threefold in E4 neurons (Fig. 4A, B). Similarly, 200 nM DEX impaired complex I activity and ATP production in E4 but not E3 neurons (Fig. S3A, B), and induced the accumulation of phospho- and total tau in E4 neurons only, as measured by immunoblotting of neuronal lysates with AT8, PHF1, and Tau5 antibodies (Fig. 4C–F). This low DEX concentration also promoted tau secretion from E4 neurons only, based on immunoblotting and ELISA of concentrated media harvested from E3 and E4 neurons (Fig. 4G, K). The increase in tau secretion was not due to compromised health or plasma membrane integrity of E4 neurons, as the levels of lactate dehydrogenase (LDH), a cytosolic protein, were similar in media from E4 vs. E3 neurons and not altered by DEX treatment (Fig. 4K). These findings indicate that GC/GR signaling and downstream tau pathogenesis are enhanced in the presence of ApoE4.

### mPTP opening drives GC-induced tau pathogenesis in E4 neurons

**Fig. 5:**
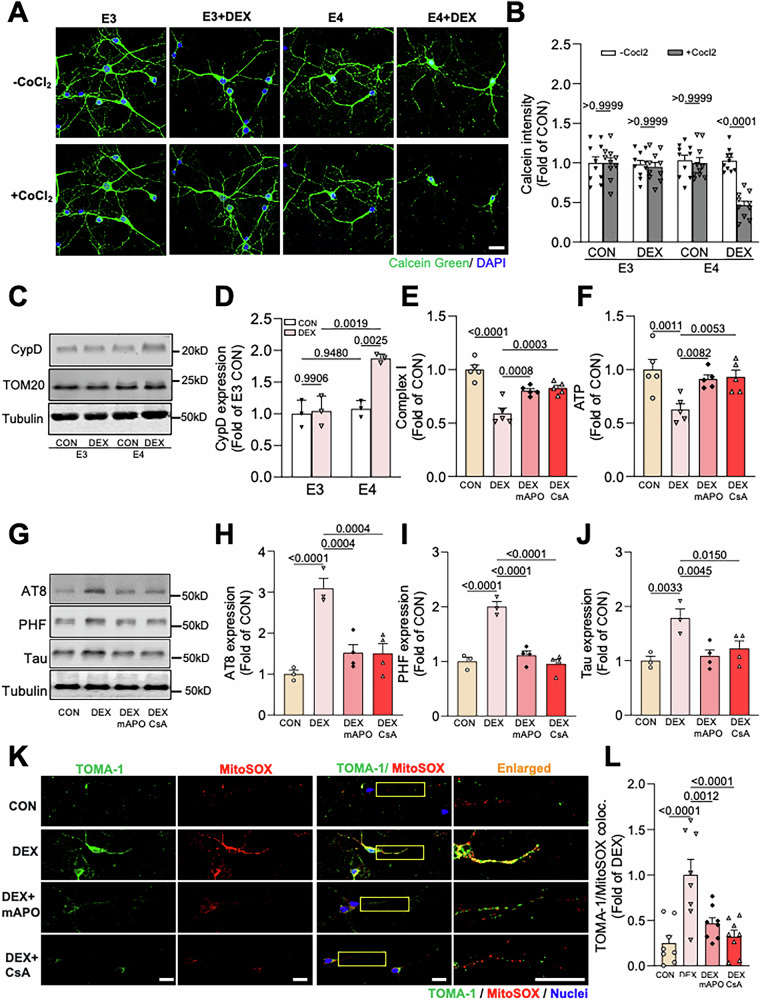
mPTP opening drives GC-induced mitochondrial dysfunction and tau pathology in E4 neurons. **A**, **B** Representative images (**A**) and quantification (**B**) of calcein green fluorescence intensity with or without CoCl_2_ in E3 and E4 hippocampal neurons treated with vehicle (CON) or dexamethasone (DEX). Scale bar = 25 µm. Intensity values are normalized to the E3 CON condition (*P* values indicated on graph; data presented as mean ± SD; two-way ANOVA with Tukey’s multiple comparisons test; *n* = 10 fields of view/condition). **C**, **D** Representative immunoblots (**C**) and quantification (**D**) of CypD and TOM20 immunoreactivity in lysates from E3 and E4 hippocampal neurons treated as indicated. Intensity values are expressed relative to tubulin and normalized to the E3 CON condition (*P* values indicated on graphs; data presented as mean ± SD; two-way ANOVA with Tukey’s multiple comparisons test; *n* = 3 samples/condition). **E**, **F** Complex I activity (**E**) and ATP levels (**F**) in E4 hippocampal neurons treated with vehicle (CON), dexamethasone (DEX), DEX + mito-apocynin (mAPO) and DEX + cyclosporin A (CsA), normalized to the CON condition (*P* values indicated on graphs; data presented as mean ± SD; one-way ANOVA with Tukey’s multiple comparisons test; *n* = 5 samples/condition). **G**–**J** Representative immunoblots (**G**) and quantification (**H**–**J**) of AT8, PHF1, and total tau (Tau5) immunoreactivity in cell lysates from E4 hippocampal neurons treated as indicated. Intensity values are expressed relative to tubulin and normalized to the CON condition (*P* values indicated on graphs; data presented as mean ± SD; one-way ANOVA with Tukey’s multiple comparisons test; *n* = 3–4 samples/condition). **K**, **L** Representative images (**K**) and quantification of TOMA-1 colocalization with MitoSOX (**L**) in E4 hippocampal neurons treated as indicated. Enlarged regions (indicated by yellow boxes) are shown in the right column. Scale bars = 50 µm (*P* values indicated on graphs; data presented as mean ± SD; one-way ANOVA with Tukey’s multiple comparisons test; *n* = 8 fields of view/condition for **L**).

We previously showed that GCs induce mitochondrial damage by promoting the opening of the mitochondrial permeability transition pore (mPTP), a channel on the inner mitochondrial membrane comprising several components, including the F_1_/F_0_ ATP synthase and the mitochondrial matrix protein cyclophilin D (CypD) [51]. Activation/opening of the mPTP is triggered by CypD [51], and we showed that GCs promote this event by transcriptionally upregulating CypD, leading to mitochondrial depolarization, overproduction of reactive oxygen species (ROS), and tau phosphorylation and oligomerization in hippocampal neurons [17]. To determine whether this mechanism is responsible for mitochondrial damage in ApoE4 neurons, we measured mPTP opening and CypD levels in 12 DIV E3 and E4 hippocampal neurons treated with vehicle or 200 nM DEX for 48 h. mPTP opening was assessed via live imaging with the Co^2+^-calcein assay [52], in which CoCl_2_-mediated quenching of calcein dye occurs upon pore opening, and CypD levels were assessed via immunoblotting of hippocampal lysates. In E3 neurons, 200 nM DEX treatment did not elicit CoCl_2_-mediated calcein quenching (Fig. 5A, B) nor the upregulation of CypD (Fig. 5C, D), demonstrating that this DEX concentration does not trigger mPTP opening. In contrast, E4 neurons treated with DEX exhibited significant CoCl_2_-mediated quenching (~50%; Fig. 5A, B) and a twofold increase in CypD expression (Fig. 5C, D), showing that even this low DEX concentration upregulates CypD and induces mPTP opening in the *APOE4* background. Since we previously found that the CypD inhibitor cyclosporin A (CsA) and the mitochondrially-targeted NADPH oxidase inhibitor mito-apocynin (mAPO) prevented GC-induced mPTP opening and downstream mitochondrial dysfunction and tau pathology in wild-type neurons [17], we tested whether these compounds had the same impact in E4 neurons. Indeed, while 200 nM DEX caused a 50% reduction in complex I activity and ATP production, both CsA (1 µM) and mAPO (1µM) prevented these effects (Fig. 5E, F) as well as the DEX-induced accumulation of total and phospho-tau in E4 neurons (Fig. 5G–J). Further, these drugs inhibited DEX-induced tau oligomerization and mROS production (Fig. 5K, L and Fig. S3C, D), demonstrating their protective role against GC-driven mitochondrial dysfunction and tau pathogenesis in E4 neurons.

### Inhibition of mPTP opening is protective against tau pathogenesis in E4 animals

**Fig. 6:**
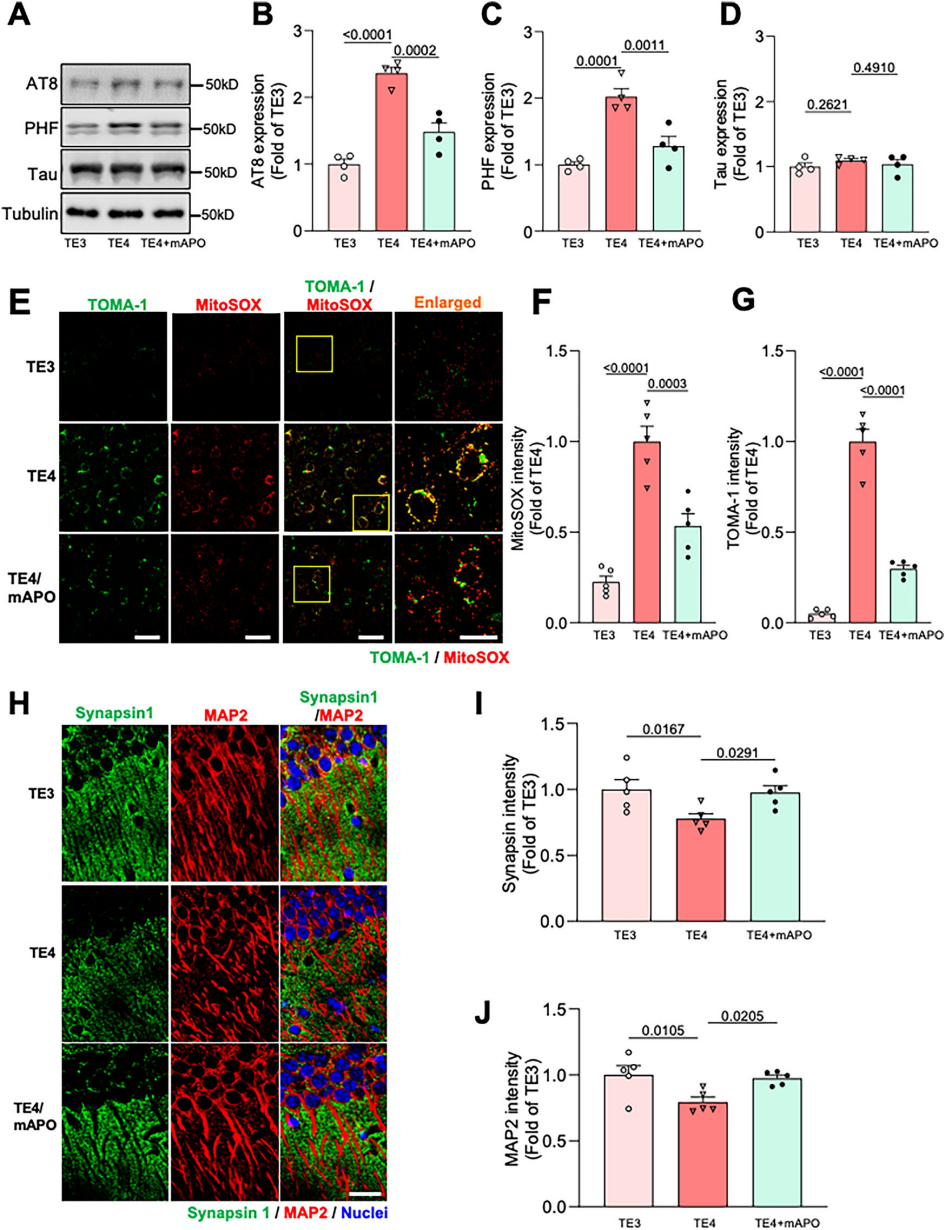
Mito-apocynin protects against mitochondrial dysfunction and tau pathology in TE4 animals. **A**–**D** Representative immunoblots (**A**) and quantification (**B**–**D**) of AT8, PHF1, and total tau (Tau5) immunoreactivity in hippocampal lysates from 3.5 to 4-month-old TE3 and TE4 mice after treatment with vehicle or mito-apocynin (mAPO). Intensity values are expressed relative to tubulin and normalized to the TE3 condition (*P* values indicated on graphs; data presented as mean ± SD; one-way ANOVA with Tukey’s multiple comparisons test; *n* = 4 samples/condition). **E**, **F** Representative images (**E**) and quantification of MitoSOX (**F**) and TOMA-1 (**G**) fluorescence intensity in hippocampal area CA1 of mice treated as indicated. Enlarged regions are shown in the right column (indicated by yellow boxes). Scale bars = 50 µm. Intensity values are normalized to TE4 controls given the absence of signal in the TE3 condition (*P* values indicated on graphs; data presented as mean ± SD; one-way ANOVA with Tukey’s multiple comparisons test; *n* = 5 mice/condition). **H**–**J** Representative images (**H**) and quantification (**I**, **J**) of Synapsin1a (green) and MAP2 (red) immunofluorescence intensity in hippocampal area CA1 of mice treated as indicated. DAPI (blue) labels nuclei. Scale bars = 50 μm. Synapsin1a and MAP2 intensity values are normalized to the TE3 condition (*P* values indicated on graphs; data presented as mean ± SD; one-way ANOVA with Tukey’s multiple comparisons test; *n* = 5 mice/condition). Each point represents an individual mouse.

The *APOE4* variant is associated with a greater risk for Alzheimer’s disease and other tauopathies compared to *APOE2* or *APOE3* [53, 54], and *APOE4* similarly exacerbates tau pathology and tau-related neurodegeneration in PS19 transgenic mice expressing the human frontotemporal dementia-linked P301S tau mutation [22]. To test whether mPTP opening plays a role in tau pathogenesis in *APOE4* carriers even in the absence of stress/high GC levels, we crossed E3 and E4 mice with PS19 tauopathy mice (hereafter called TE3 and TE4) and administered vehicle or mAPO (3 mg/kg; daily i.p. injection) to 1–1.5-month-old animals prior to the onset of any pathological changes. Ten weeks later, we euthanized the animals, harvested their brain tissue, and assessed tau-related pathology in the hippocampus. In hippocampal tissue, we observed a twofold increase in phosphorylated tau levels in samples from TE4 versus TE3 animals (Fig. 6A–D), as well as dramatically elevated oligomeric tau and associated mROS in neurons of area CA1 (Fig. 6E–G). Administration of mAPO significantly attenuated these phenotypes (Fig. 6A–G). Phosphorylated and oligomeric tau redistributes from axons into the somatodendritic compartment, inducing synaptic and neuronal loss that is thought to underlie cognitive decline in Alzheimer’s and other tauopathies [55, 56]. To determine whether mAPO also mitigates this downstream impact of tau pathology, we examined synapse density in hippocampal area CA1. Immunostaining with antibodies against the presynaptic marker synaptophysin and the somatodendritic marker MAP2 revealed that the density of synapses and MAP2^+^ dendrites was significantly decreased in control TE4 versus TE3 animals, and both phenotypes were prevented by mAPO (Fig. 6H–J). As anticipated, treatment with mAPO also normalized mitochondrial dysfunction in TE4 animals (Fig. S3E, F). These findings indicate that mitochondrial dysfunction, and mPTP opening in particular, plays a key role in ApoE4-mediated tau pathogenesis and associated synapse loss in the hippocampus.

**Fig. 7:**
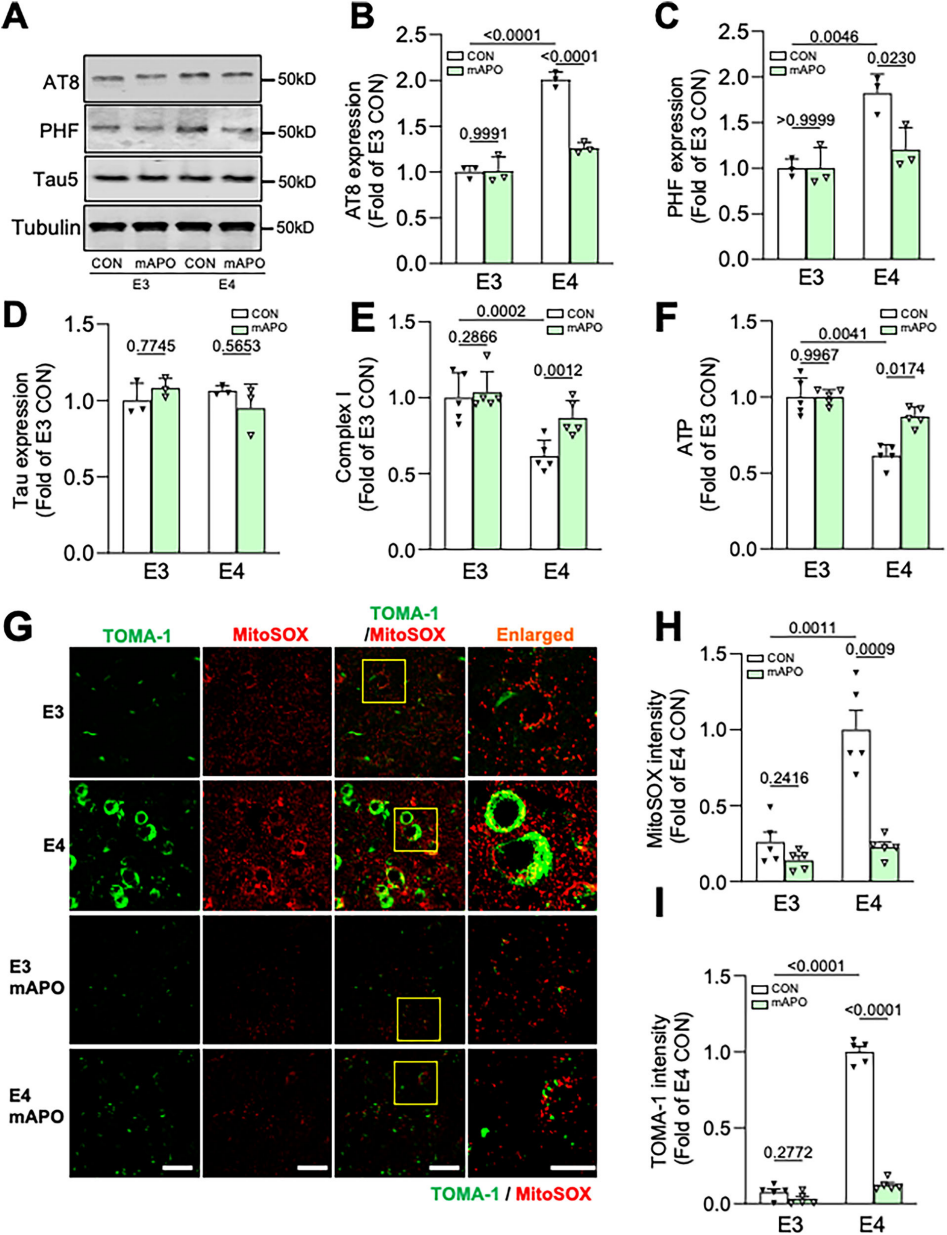
Mito-apocynin prevents mitochondrial dysfunction and tau pathology in aging E4 animals. **A**–**D** Representative immunoblots (**A**) and quantification (**B**–**D**) of AT8, PHF1, and total tau (Tau5) immunoreactivity in hippocampal lysates from 15 to 16-month-old E3 and E4 mice treated for 15 days with vehicle (CON) or mito-apocynin (mAPO). Intensity values are expressed relative to tubulin and normalized to the E3 CON condition (*P* values indicated on graphs; data presented as mean ± SD; two-way ANOVA with Tukey’s multiple comparisons test; *n* = 3 mice/condition). **E**, **F** Complex I activity (**E**) and ATP levels (**F**) in hippocampal tissues treated as indicated, normalized to the E3 CON condition (*P* values indicated on graphs; data presented as mean ± SD; one-way ANOVA with Tukey’s multiple comparisons test; *n* = 5–6 mice/condition). **G**–**I** Representative images (**G**) and quantification (**H**, **I**) of MitoSOX and TOMA-1 fluorescence intensity in hippocampal area CA1 of mice treated as indicated. Enlarged regions are shown in the right column (indicated by yellow boxes). Scale bars = 50 µm. Intensity values are normalized to the E4 CON condition (*P* values indicated on graphs; data presented as mean ± SD; two-way ANOVA with Tukey’s multiple comparisons test; *n* = 5 mice/condition).

While the previous experiment shows that mAPO confers remarkable protection against ApoE4-induced tau pathology, it was conducted in a mouse model overexpressing pathogenic P301S tau, a non-physiological condition. To study the efficacy of mAPO in a condition more relevant to human Alzheimer’s disease, we utilized aging E3 and E4 animals. In particular, we administered vehicle or mAPO (3 mg/kg, i.p. injection) to 15–16-month-old E3 and E4 animals for 15 days, then harvested brain tissue to measure tau phosphorylation/oligomerization and mitochondrial function as described above. In contrast to 9–10-month-old E4 (Fig. 1) and 15–16-month-old E3 mice, the 15–16-month-old E4 animals exhibited a twofold increase in the levels of phosphorylated tau in hippocampal tissue as measured by AT8 and PHF1 antibodies, with no change in total tau (Fig. 7A–D). Treatment with mAPO had no impact on total or phospho-tau levels in E3 brain tissue, but normalized phospho-tau levels in E4 tissue to those of the E3 controls (Fig. 7A–D). Similarly, complex I activity and ATP production in aging E4 animals were reduced by 50% compared to E3 animals, and mAPO normalized both of these to the level of E3 control animals without impacting baseline mitochondrial function in the E3 background (Fig. 7E, F). Levels of mROS and oligomeric tau were likewise dramatically increased in aging E4 vs. E3 animals and rescued in E4 animals by mAPO (Fig. 7G–I).

**Fig. 8:**
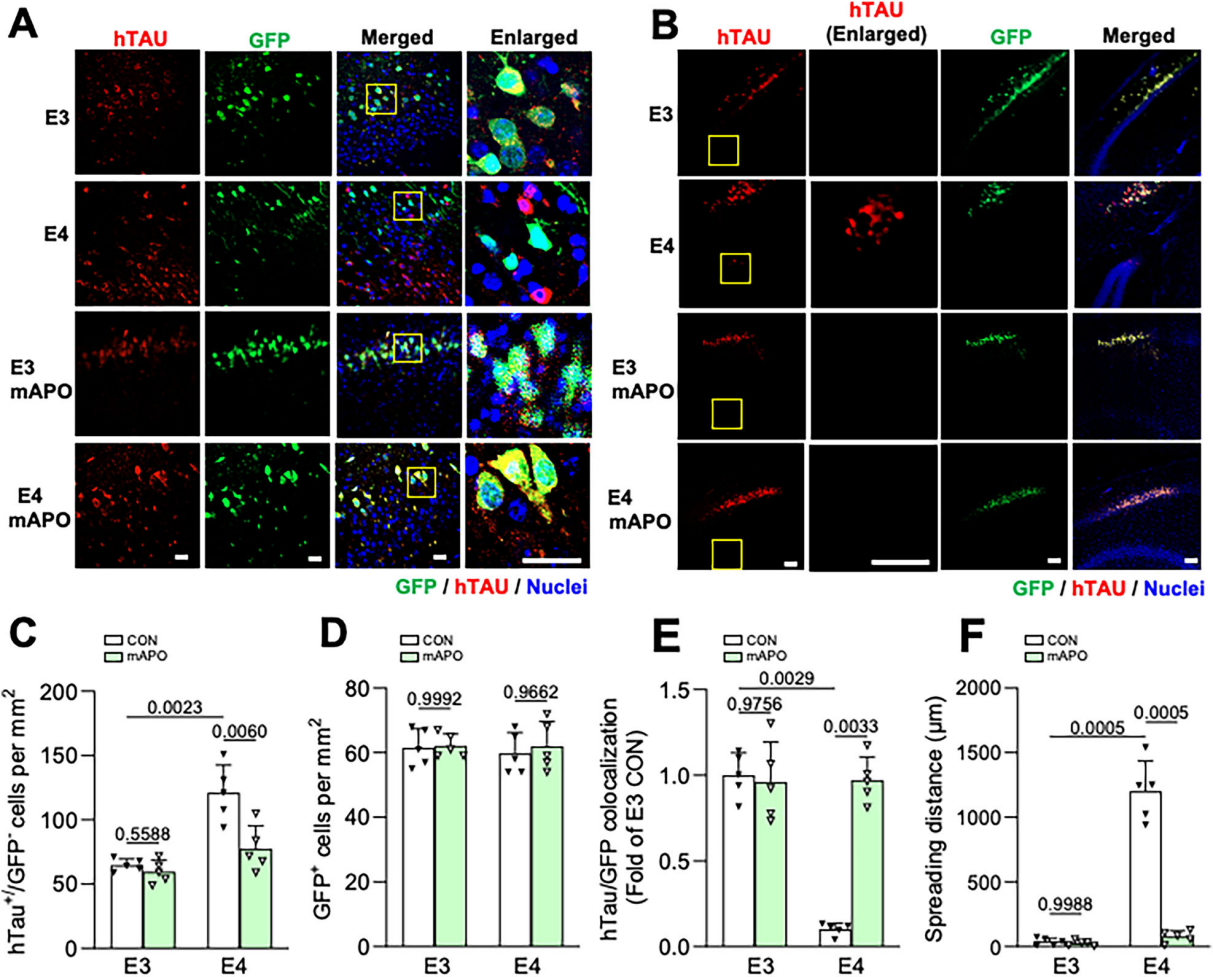
Mito-apocynin prevents tau spreading in aging E4 animals. **A** Representative images showing hTau (red) and GFP (green) in CA1 neurons of from 15 to 16-month-old E3 and E4 mice treated with vehicle (CON) or mito-apocynin (mAPO). Nuclei are stained with DAPI (blue). The right column shows enlarged regions (indicated by yellow boxes). Scale bars, 50 µm. **B** Representative images depicting the spreading of hTau (red) from GFP^+^ cells near the injection site in mice treated as indicated. Scale bars = 200 µm. **C** Quantification of hTau^+^/GFP^−^ cells per mm^2^ in mice treated as indicated. **D** Quantification of GFP^+^ cells per mm^2^ in mice treated as indicated. **E** Quantification of the hTau/GFP colocalization ratio in each condition, normalized to E3 CON condition. **F** Quantification of Tau spreading distance (mm) for each condition (*P* values indicated on graphs; data presented as mean ± SD; two-way ANOVA with Tukey’s multiple comparisons test; *n* = 5 mice/group). Each point represents an individual mouse.

Finally, we examined the impact of mAPO on tau propagation, shown to be enhanced in the *APOE4* genotype [57, 58]. Here, 15–16-month-old animals were administered vehicle or mAPO for one week prior to AAV injection of AAV.CBA.eGFP.2 A.P301L-Tau in hippocampal area CA1, and tau spreading was assessed two weeks later as described above. In contrast to tau spreading in the younger (9–10-month-old) mice, for which no significant difference was observed between genotypes (Fig. 1), spreading in 15–16-month-old mice was significantly increased for E4 versus E3 animals (Fig. 8A, B). Specifically, E4 hippocampi had increased numbers of hTau^+^/GFP^−^ neurons per mm^2^, decreased GFP/hTau colocalization, and increased hTau spreading distance ( > 1000 μm for E4 vs. <100 mm for E3) compared to E3 hippocampi (Fig. 8C, E, F), while AAV transduction efficiency (GFP^+^ cells/mm^2^) was equivalent between genotypes (Fig. 8D). Administration of mAPO treatment completely normalized the enhanced spreading in E4 brains, demonstrating that mitochondrial dysfunction, and mPTP opening in particular, plays an important role in driving tau propagation in *APOE4* carriers.

## Discussion

Whether and how *APOE4*, the most common genetic Alzheimer’s disease risk factor, confers vulnerability to chronic stress and contributes to stress-related brain pathology has been unclear. In the current study, we find that GC-induced mitochondrial damage and ROS production, as well as tau hyperphosphorylation, oligomerization, and spreading, is exacerbated in *APOE4* humanized knockin mice compared to their *APOE3* counterparts, consistent with the reported vulnerability of *APOE4* carriers to stress [10, 14, 20, 59–61]. Moreover, E4 animals exhibit mitochondrial dysfunction and enhanced GR activation at baseline, indicating that these underlying factors promote tau pathology and Alzheimer’s progression in the presence and absence of external stressors. Mechanistically, we show that the mPTP is a key driver of mitochondrial damage and tau pathology in the E4 background, and that mPTP inhibition is protective against both features in aging E4 mice. These findings shed light on the mechanisms of *APOE4*-mediated stress vulnerability and Alzheimer’s pathophysiology.

Multiple studies show a link between *APOE4* and mitochondrial dysfunction [18–21, 42, 45–47], but the mechanisms by which ApoE4 causes this dysfunction remain unclear. In addition to its role as a secreted protein, ApoE localizes to mitochondria and mitochondria-associated endoplasmic reticulum membranes (MAMs) and is reported to interact with a number of mitochondrial and MAM proteins [47, 62], suggesting a role in these compartments. Moreover, C-terminally truncated ApoE4 fragments, the product of ApoE proteolytic cleavage in the neuronal cytosol [63], were found to bind and inhibit the activity of mitochondrial respiratory chain proteins, leading to mitochondrial dysfunction [64]. These proteolytic fragments can also induce MAM formation and mitochondrial calcium overload, promote mitochondrial fission and fragmentation, and induce transcriptional changes in the nucleus [45, 64, 65], all of which may contribute to mitochondrial dysfunction in neurons. In cardiomyocytes, ApoE bound to a specific form of low-density lipoprotein was also observed to colocalize with the voltage-dependent anion-selective channel 1 (VDAC1) on the outer mitochondrial membrane and induce mPTP opening [66], indicating a role for ApoE in regulating mPTP opening via VDAC1. Additional research will be required to determine whether this function is specific to, or enhanced by, the E4 variant.

In our previous study conducted in wild-type mice, we showed that GC-induced mitochondrial damage precipitates tau pathology [17], and we see an augmented version of this phenomenon in *APOE4* animals. Precisely how mitochondrial damage drives tau pathogenesis is unknown, but several potential mechanisms have been implicated. These include mROS-mediated activation of kinases (e.g., GSK3β, CDK5, ERK1/2) [67] that may stimulate tau hyperphosphorylation, mROS direct oxidation of specific tau methionine residues, a modification reported to induce tau cleavage and aggregation [68], and leakage of the mitochondrial succinylating enzyme dihydrolipoamide succinyltransferase (DLST) into the cytoplasm, which can induce the succinylation and subsequent aggregation of tau [69]. There is also increasing evidence that ATP functions as a biological hydrotrope to maintain protein solubility in the cytosol, and that reduced levels of cytosolic ATP (e.g., resulting from mitochondrial dysfunction) contribute to the aggregation of intrinsically-disordered proteins such as tau [70, 71]. All of these mechanisms could play a role in tau aggregation specifically, and more generally in the loss of proteostasis during chronic stress, aging, and Alzheimer’s pathogenesis, and thus merit future investigation.

Notably, tau pathology appears to lag mitochondrial dysfunction by several months in E4 mice. While 9–10-month-old E4 animals exhibit signs of mitochondrial damage (i.e., increased ROS levels, decreased complex 1 activity and ATP production) without any increase in tau hyperphosphorylation, oligomerization, or spreading compared to their E3 counterparts (Figs. 1, 2), 15–16-month-old E4 animals exhibit both mitochondrial dysfunction and tau pathology (Figs. 7, 8). Such a lag could indicate that additional, mitochondria-independent mechanisms (e.g., degradative pathway inhibition, up-/downregulation of tau kinases and phosphatases) contribute to tau pathogenesis in the E4 background, and that these become more pronounced over time. Alternatively, our findings may not reflect a lag between these events, but rather enhanced sensitivity of the methods used to detect mitochondrial damage (i.e., EPR spectroscopy, commercial kits) versus tau pathology (i.e., immunostaining), thus facilitating earlier detection of mitochondrial dysfunction. Future experiments will distinguish between these possibilities.

An unexpected observation from this study is that GR activation, and GC-mediated signaling, appear to be enhanced in the *APOE4* background. Several factors impact GR activation, including its chaperones, phosphorylation, and GC bioavailability [48]. Here, we show increased levels of the major GR co-chaperones Hsp90, Hsp70, and FKBP51 in brain tissue from E4 mice (Fig. 3). This finding is consistent with another study showing upregulation of HSP70 and HSP90 transcripts in TE4 mice and their downregulation following neuron-specific *APOE4* deletion [23]. It was recently reported that hormone binding to GR is accelerated by Hsp90 [49], indicating a potential mechanism by which increased Hsp90 levels could enhance GR activation in E4 neurons during GC exposure. However, other mechanisms are likely responsible for the observed activation of GR/GC signaling pathways in the brains of E4 animals in the absence of exogenous/elevated GC levels (Fig. 3 and Fig. S2). For instance, ApoE4 may alter the levels of enzymes involved in cortisol (for humans)/corticosterone (for mice) bioavailability. These include 11β-hydroxysteroid dehydrogenases (11β-HSDs), which convert inactive cortisone (or 11-dehydrocorticosterone, in the case of mice) to cortisol/corticosterone [48]. Interestingly, a recent pre-print implicates local activation of 11β-HSD1 in *APOE4* carrier-specific atrophy of the entorhinal cortex, associated with progression from mild cognitive impairment to AD in *APOE4* carriers [72]. Alternatively, ApoE4 could up- or downregulate the levels of kinases and/or phosphatases that mediate GR phosphorylation (e.g., CDK2, JNK, p38 MAPK, PP5) [48], leading to higher basal GR activation. We hypothesize that any such ApoE4-mediated changes also lower the threshold for GR activation by GCs and intensify with aging, leading to higher reactivity to stress/high GC levels in aged *APOE4* carriers. Indeed, chronic stress-induced depression is exacerbated in both aged humans and aged mice carrying *APOE4* [10, 20, 61].

Given the lack of effective Alzheimer’s disease therapies for *APOE4* carriers, who are highly susceptible to the most serious side effects of amyloid-clearing immunotherapy (i.e., amyloid-related imaging abnormalities with edema or hemorrhages, referred to as ARIA-E or ARIA-H) [73], an important finding from our work is that inhibition of the mPTP is protective against ApoE4-mediated tau pathogenesis. Indeed, we show that mAPO prevents tau pathology when given for 2.5 months to young (~1.5-month-old) TE4 mice prior to the onset of tauopathy-related phenotypes, and when given for a more limited, 15-day period to aging (15–16-month-old) E4 mice. In the young TE4 animals, mAPO administration not only protects against mitochondrial dysfunction and tau hyperphosphorylation/oligomerization in the hippocampus, but also prevents synapse loss, indicating amelioration of the synaptotoxic effects of pathogenic tau associated with cognitive impairment. Similarly, mAPO administration to aging E4 animals normalizes their mitochondrial function, tau phosphorylation/oligomerization, and tau spreading to the levels of E3 control animals, suggesting that mPTP inhibition in middle age can effectively counteract ApoE4-driven pathology. These findings could indicate that the precipitating event for stress/GC-induced mitochondrial dysfunction (i.e., transcriptional upregulation of CypD leading to mPTP opening) is conserved in TE4 and aging E4 animals, or that other mechanisms stimulate mitochondrial damage/mPTP opening and mAPO prevents the downstream effects of this damage by inhibiting further mROS production and mPTP opening. Both pathogenic tau and ApoE4 are reported to induce mitochondrial damage through multiple mechanisms, including disruption of mitochondrial transport, fission/fusion, and membrane potential for tau [74] and interference with MAMs, VDAC, and respiratory chain proteins for ApoE4 [64–66]. Thus, additional studies are needed to uncover the specific mechanisms of mitochondrial dysfunction in each of these contexts.

While our experiments here have focused on mitochondrial dysfunction and tau pathology, the other major Alzheimer’s pathomechanisms, amyloid-beta (Aβ) production and neuroinflammation, are also augmented by stress/GCs and ApoE4, and recent studies suggest that they would also be mitigated by mPTP inhibition. Indeed, Aβ was shown to interact with the mitochondrial membrane and trigger opening of the mPTP, while CypD inhibitors or knockout have protective effects against Aβ-induced toxicity in animal models [75, 76]. In addition, mPTP opening is reported to trigger activation of pro-inflammatory pathways through the release of mitochondrial DNA into the cytosol [77–79], and CypD inhibitors or knockout reduce pro-inflammatory signaling in animal models of acute inflammation and Alzheimer’s disease [76, 80, 81]. Other studies show that mAPO administration or CypD inhibition/knockout are protective against neurodegeneration in animal models of Alzheimer’s and Parkinson’s diseases and multiple sclerosis [75, 82–87], demonstrating the broader therapeutic promise of targeting the mPTP. Although the specific CypD inhibitor CsA doesn’t cross the blood-brain-barrier and thus cannot be readily used to target the central nervous system [88], other inhibitors are currently under development [76, 89] and represent an exciting new category of drugs for the treatment of neurodegenerative disease.

In summary, we find that mitochondrial dysfunction underlies chronic stress- and ApoE4-induced tau phosphorylation, oligomerization, and spreading, and that inhibition of the mPTP prevents these effects in *APOE4* carriers. This strategy may represent a viable therapeutic option for preventing stress- and ApoE4-mediated neurodegeneration in Alzheimer’s disease.

## Supplementary information

Supplemental Figure Legends

Figure S1

Figure S2

Figure S3

Figure S4

Supplemental Table 1

## Data Availability

The data that support the findings of this study, generated and analyzed in this study, are available from the corresponding author upon reasonable request.
