# Supplementary material for: APOE4 exacerbates glucocorticoid stress hormone-induced tau pathology via mitochondrial dysfunction
Source: Cell Death Dis. 2026 Mar 27;17(1):419. doi: 10.1038/s41419-026-08543-1 (PMC13150023; doi:10.1038/s41419-026-08543-1)
Supplement: Supplementary file 1 — Supplemental Figure Legends [file 41419_2026_8543_MOESM1_ESM.docx]

**Supplemental Material**

**Supplemental Figure Legends**

**Figure S1: Systemic effects of dexamethasone and purity of mitochondrial, cytosolic and nuclear fractionation.** (**A**) Quantification of corticosterone levels measured by ELISA in serum from 9-10-month-old E3/E4 mice treated with vehicle (CON) or dexamethasone (DEX)(*P-*values indicated on graphs; unpaired *t*-test; n=5 mice per group). (**B**) Quantification of body weight loss in the indicated mice (**P_E3CON VS. E3DEX_ <0.01 (days 13-15), **P_E4CON VS. E4DEX_ <0.01 (days 13-15), #P_E3CON VS. E3DEX_ <0.05 (days 11-12), #P_E4CON VS. E4DEX_ <0.01 (days 10-12); two-way ANOVA with multiple comparisons; n=10 mice/condition). All data presented as mean ± SD. (**C**) Immunoblots of mitochondrial, cytosolic, and nuclear fractions probed with mitochondrial marker TOM20, nuclear marker KDM1/LSD1, and cytosolic marker tubulin, indicating high purity and low contamination of each fraction.

**Figure S2: Analysis of GC-relevant gene expression changes in APOE4 vs APOE3 mice.** (**A**) Clustering after principal component analysis (PCA) revealed 24 total clusters, visualized using UMAP (top graph) and the first two principal components (bottom graph). (**B**) Feature plot shows expression of dentate gyrus markers Stxbp6 and Dgkh, indicating that clusters 1 and 2 are the main dentate gyrus clusters (and the focus of the following analyses). (**C**) Dot plot shows expression of several other dentate gyrus markers. (**D-E**) Volcano plot produced after differential expression gene analysis on cluster 1 (**D**) and cluster 2 (**E**),with the top 15 genes labeled and false discovery rate of 0.20. (**F-G**) Functional ontology pathways from ShinyGO v0.82 after entering the list of both cluster 1 and 2 upregulated (**F**) or downregulated genes (**G**). The background list for both functional pathway enrichments was the same 27,500 genes initially detected from the raw data and referenced against the Mus musculus (GRCm39) species reference genome.

**Figure S3:** **APOE4 carriers exhibit mitochondrial dysfunction and tau pathology that is rescued by inhibitors of mPTP opening.** (**A**-**B**) Complex I activity (**A**) and ATP levels (**B**) in E3/E4 hippocampal neurons treated with vehicle (CON) or dexamethasone (DEX), normalized to the E3 CON condition (*P*-values are indicated on the graphs; data presented as mean ± SD; two-way ANOVA with Tukey’s multiple comparisons test; n=5 samples/condition). (**C**-**D**) Quantification of TOMA-1 (**C**) and MitoSOX (**D**) fluorescence intensity in primary 14 DIV E4 hippocampal neurons treated with vehicle (CON), DEX, DEX + mito-apocynin (mAPO), or DEX + cyclosporin A (CsA). Intensity values are normalized to the DEX condition (*P-*values indicated on graphs; data presented as mean ± SD; one-way ANOVA with Tukey’s multiple comparisons test; n=8 fields of view/condition). (**E-F**) Complex I activity (**E**) and ATP levels (**F**) in hippocampal tissue of 3.5-4-month-old TE3/TE4 mice treated with vehicle (CON) or mito-apocynin (mAPO), normalized to the TE3 condition (*P-*values indicated on graphs; data presented as mean ± SD; one-way ANOVA with Tukey’s multiple comparisons test; n=5-6 samples/condition). Each point represents an individual mouse.

**Figure S4: Original data.** Full immunoblots for all figures. Rectangular boxes indicate the regions shown in the indicated figures. Proteins are labeled on the left and sizes (kD) on the right.
