# Supplementary figures and images for: APOE4 exacerbates glucocorticoid stress hormone-induced tau pathology via mitochondrial dysfunction
Source: Cell Death Dis. 2026 Mar 27;17(1):419. doi: 10.1038/s41419-026-08543-1 (PMC13150023; doi:10.1038/s41419-026-08543-1)

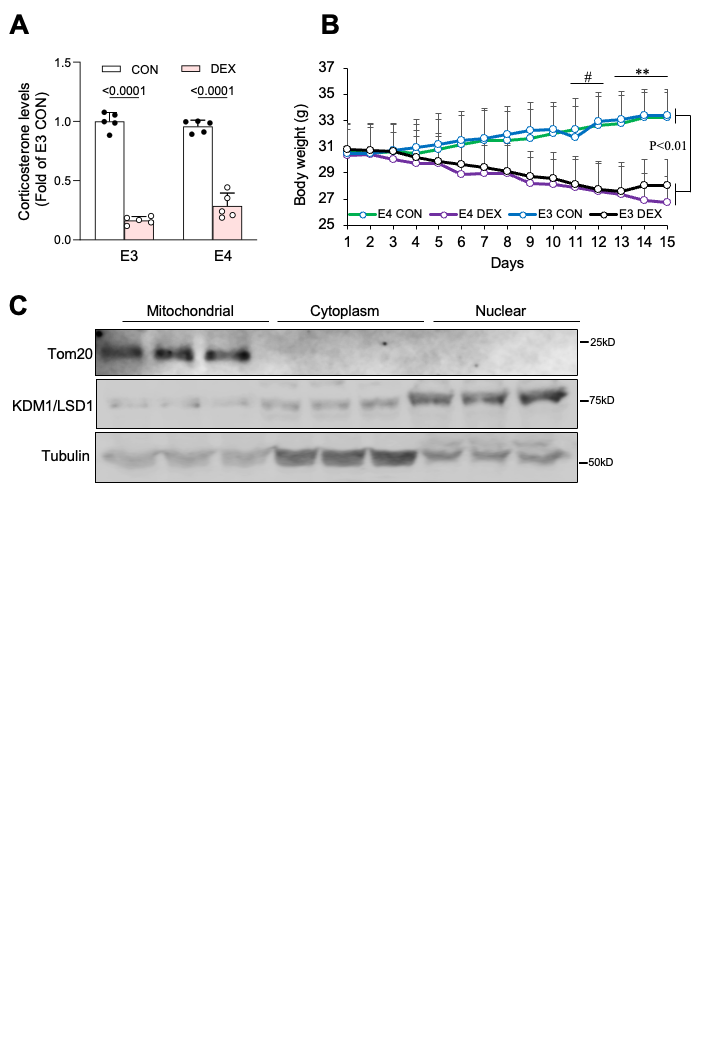

Supplement: Supplementary file 2 — Figure S1 [file 41419_2026_8543_MOESM2_ESM.tif]

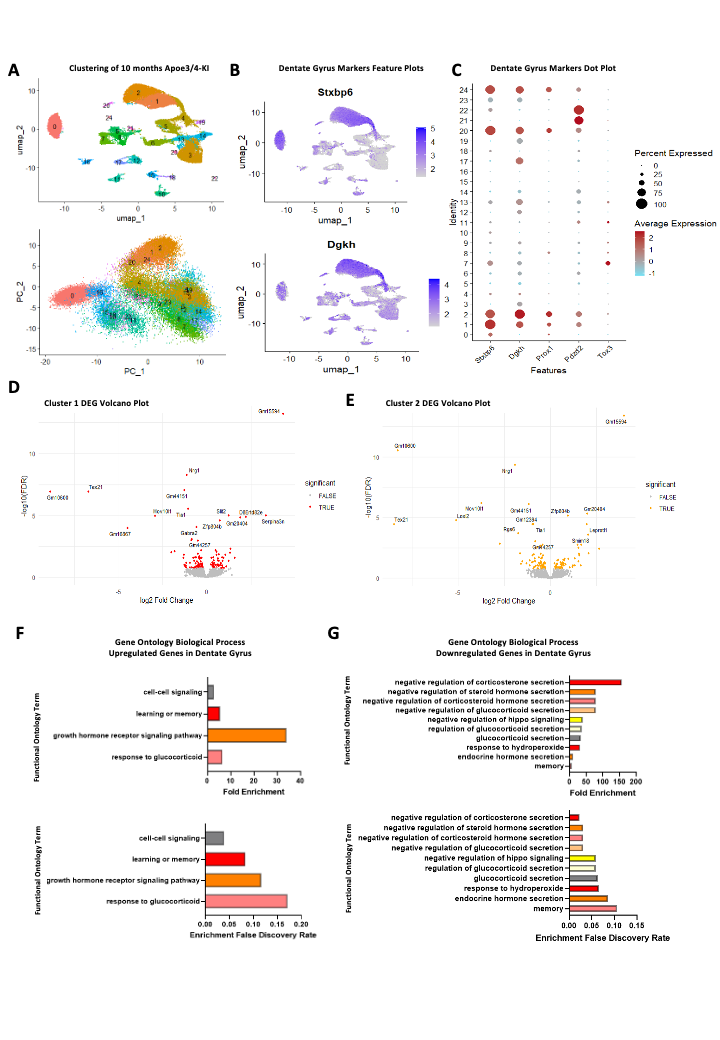

Supplement: Supplementary file 3 — Figure S2 [file 41419_2026_8543_MOESM3_ESM.tif]

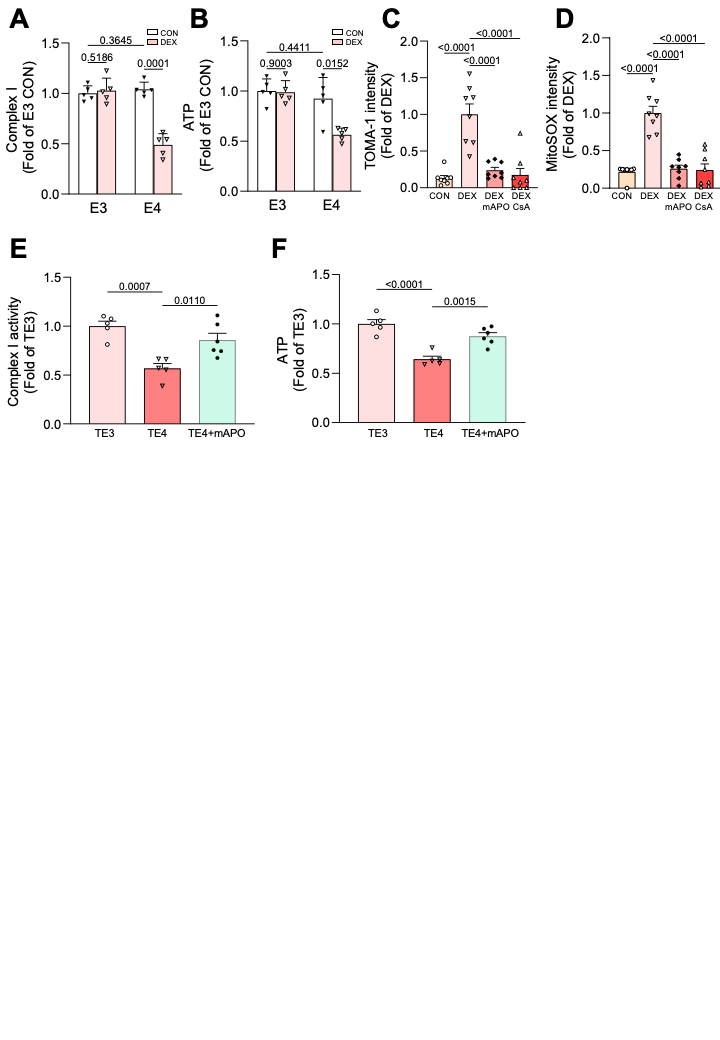

Supplement: Supplementary file 4 — Figure S3 [file 41419_2026_8543_MOESM4_ESM.tif]

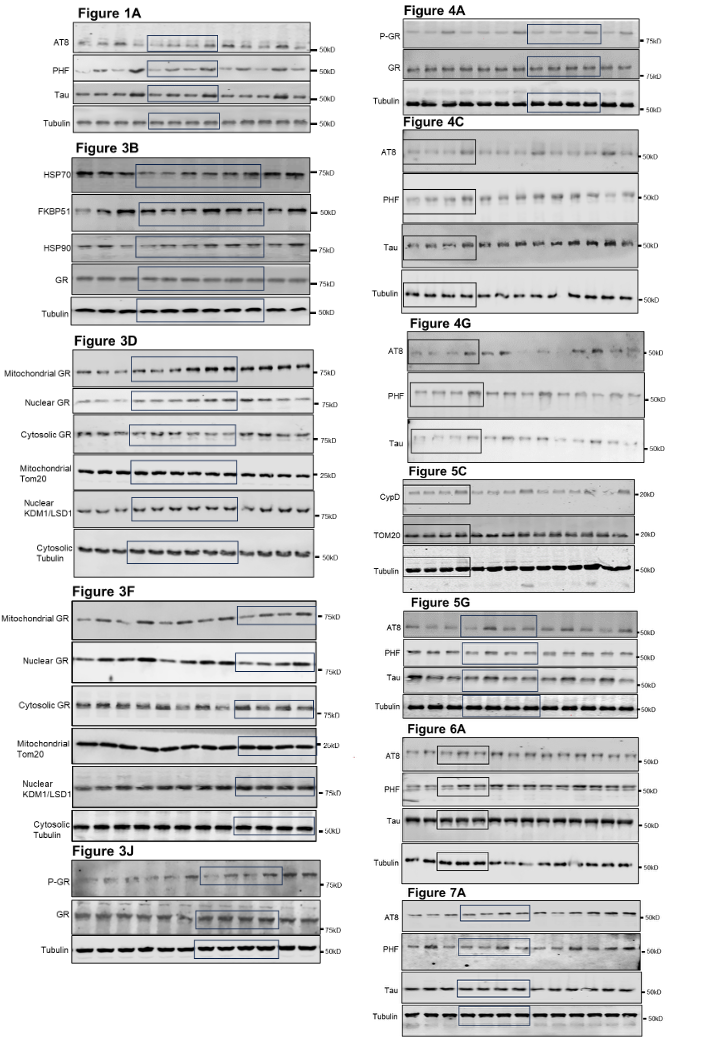

Supplement: Supplementary file 5 — Figure S4 [file 41419_2026_8543_MOESM5_ESM.tif]
